# Single-cell transcriptome analysis profiling lymphatic invasion-related TME in colorectal cancer

**DOI:** 10.1038/s41598-024-59656-6

**Published:** 2024-04-17

**Authors:** Liping Wang, Liming Ma, Zhaona Song, Li Zhou, Kexin Chen, Xizi Wang, Zhen Liu, Baozhong Wang, Chen Shen, Xianchao Guo, Xiaodong Jia

**Affiliations:** 1 Department of Geriatrics, Liaocheng People’s Hospital, Liaocheng, 252000 Shandong China; 2 Harbin Inji Technology Co., Ltd., Harbin, 150060 Heilongjiang China; 3 Joint Laboratory for Translational Medicine Research, Liaocheng People’s Hospital, Liaocheng, 252000 Shandong China; 4 Department of Oncology, Liaocheng People’s Hospital, Liaocheng, 252000 Shandong China; 5 Department of Data and Information, Children’s Hospital, Zhejiang University School of Medicine, Hangzhou, 310052 Zhejiang China; 6 Beijing Easyresearch Technology Limited, Beijing, 100049 China

**Keywords:** Cancer microenvironment, Tumour biomarkers, Lymphatic system

## Abstract

Lymphatic invasion (LI) is extremely aggressive and induces worse prognosis among patients with colorectal cancer (CRC). Thus, it is critical to characterize the cellular and molecular mechanisms underlying LI in order to establish novel and efficacious therapeutic targets that enhance the prognosis of CRC patients. RNA-seq data, clinical and survival information of colon adenocarcinoma (COAD) patients were obtained from the TCGA database. In addition, three scRNA-seq datasets of CRC patients were acquired from the GEO database. Data analyses were conducted with the R packages. We assessed the tumor microenvironment (TME) differences between LI+ and LI− based scRNA-seq data, LI+ cells exhibited augmented abundance of immunosuppression and invasive subset. Marked extracellular matrix network activation was also observed in LI+ cells within SPP1+ macrophages. We revealed that an immunosuppressive and pro-angiogenic TME strongly enhanced LI, as was evidenced by the CD4+ Tregs, CD8+ GZMK+, SPP1+ macrophages, e-myCAFs, and w-myCAFs subcluster infiltrations. Furthermore, we identified potential LI targets that influenced tumor development, metastasis, and immunotherapeutic response. Finally, a novel LIRS model was established based on the expression of 14 LI-related signatures, and in the two testing cohorts, LIRS was also proved to have accurate prognostic predictive ability. In this report, we provided a valuable resource and extensive insights into the LI of CRC. Our conclusions can potentially benefit the establishment of highly efficacious therapeutic targets as well as diagnostic biomarkers that improve patient outcomes.

## Introduction

Colorectal cancer (CRC) ranks third in the world in terms of incidence, and it is the second contributor to cancer-related mortality^1^. Metastasis is the major cause of death in patients with CRC, and lymphatic invasion (LI) is an early metastatic event that serves as a stand-alone risk factor influencing the prognosis of CRC patients^2^. Thus far, multiple attempts have been made to elucidate the LI-associated genes and signaling pathways within CRC. Shi et al. suggested that the genetic and transcriptional alterations in lymphatic invasion were associated with tumor microenvironment (TME)^3^. Zhang et al.^4^ suggested that lymph node metastasis-related signature was associated with immune infiltrating cells. Sarah et al.^5^ suggested that the epithelial cell-specific p53 deletion markedly enhanced cancer invasive and lymph node metastasis. These studies suggested LI in CRC were typically controlled by an intricate and dynamic cellular network within the TME. It is clear that an extensive knowledge of the complex TME (including heterogeneous cancer cells, diverse infiltrating immune, as well as stromal cells) which modulates LI in CRC is crucial for the regulation of CRC development, metastasis, and prognosis, as well as LI-based therapies.

However previous studies primarily examined bulk RNA-Seq data or genetic aberrations, and analysis using bulk RNA-seq seems not suitable to reveal the dynamic status of various cells and their interactions involving in the progression of tumor. In this regard, Single-cell RNA sequencing (scRNA-seq) is a robust and innovative technology for discerning cellular and molecular heterogeneities of CRC samples^6–10^. One recent scRNA-seq study demonstrated strong tumor-associated macrophage (TAM) heterogeneity among CRC tissue^7^. Additionally, scRNA-seq also defines a continuum of cellular states and compositional alterations in the malignant polyps transformation to colorectal cancer^11^. Herein, we aimed to validate and further evaluate the heterogeneous cellular and transcriptomic profiles of LI in CRC. To do this, we extracted CRC scRNA-seq datasets from the GEO database, namely, those with accession numbers GSE200997, GSE166555, and GSE201348^11–13^. To minimize batch effect among the acquired scRNA-seq datasets, we independently assessed individual datasets to obtain 7 major cellular components. Next, to fine-tune our exploration of the TME, we combined the immunocyte and fibroblast cell sets of tumor tissues from the aforementioned 3 datasets to generate second-stage clustering. Lastly, to identify cell subpopulations that promote LI, we employed scissor^14^ to associate cells with LI, forming a relative concept. Thus, we utilized scRNA-seq to not only identify specific immunocyte and fibroblast sub populations that promote LI in CRC patients, but also recognized certain invasive genes as potential therapeutic targets to enhance outcomes of the same patients. Meanwhile, we also further verified the enrichment of particular sub-cell populations using TCGA-COAD-derived bulk RNA-seq. Using the infiltrated gene expressions, we next stratified TCGA-COAD patients into 3 subgroups with different carcinogenic profiles. We revealed that the patient clinical outcome, immune cell score, and activated functional networks were strongly distinct among the 3 subcategories. Thus, we identified potential therapeutic targets for enhancement of CRC patient outcomes. In addition, herein, we provided valuable mechanistic insights into lymphatic infiltration as well as establishment of personalized therapies for CRC patients.

## Results

### Identification of major cell types

**Figure 1:**
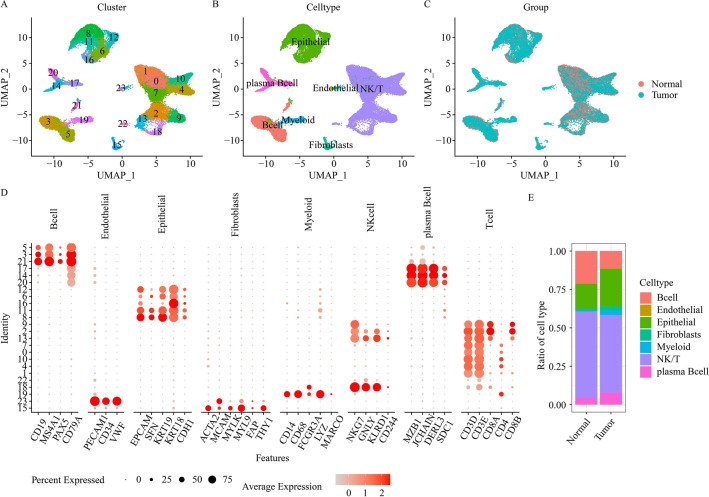
A summary of the single cells in CRC patients, and recognition of primary cell types in the GSE200997 dataset. (**A**) UMAP plot depicting single cells (colored according to cell cluster). (**B**) UMAP plot depicting single cells (colored according to cellular type). (**C**) UMAP plot depicting single cells (colored according to sample origins, either tumor versus normal samples). (**D**) Dot plot illustrating representative marker genes across all cellular clusters. Dot size indicates fraction of specific gene-expressing cells. Color intensity indicates relative specific gene expressions. (**E**) Stacked bar chart depicting 7 major cellular type contents in individual tumor or normal samples.

To systematically evaluate TME significance in CRC patients, we obtained scRNA-seq data from 3 datasets, encompassing 35 CRC patients and 4 healthy controls, with 34 tumor and 23 non-tumor samples. Among the 23 non-tumor samples, 19 were adjoining non-malignant tissues from CRC patients. To minimize batch effect among the scRNA-seq datasets, we separately assessed individual dataset. Following low-quality cell filtration, we acquired 42,696, 68,702, and 118,904 single cells, and generated unsupervised clustering of 24, 31, and 39 clusters, respectively for the GSE200997, GSE166555 and GSE201348 CRC datasets (Fig. 1A, Supplementary Figs. 1A and 2A). The aforementioned clusters were, in turn, separated into 7 primary cell components, according to their associated canonical markers, and these were, fibroblasts (harboring ACTA2, MCAM, MYLK, MYL9, FAP and THY1), epithelial cells (harboring EPCAM, SFN, KRT19, KRT18 and CDH1), myeloid cells (harboring CD68, CD14, FCGR3A,LYZ and MARCO), endothelial cells (harboring VWF, PECAM1 and CD34), NK/T cells (harboring NKG7, GNLY, KLRD1, CD24, CD3,CD4 and CD8), B cells (harboring CD19, MS4A1,PAX5 and CD79A) and plasmaB cells (harboring MZB1, JCHAIN, JCHAIN and SDC1) (Fig. 1B,D, Supplementary Figs. 1B,D and 2B,D). Figure 1C, Supplementary Figs. 1C and 2C illustrate the cell-type compositions and tissue origins of each dataset. Based on our observation, cells from tumor samples were integrated with cells from non-tumor samples. This indicated no marked batch effects among different samples during clustering. To better elucidate cellular clustering, we performed cell proportion analysis. We revealed un-even distribution of the relative abundances of the 7 primary cellular populations between tumor and non-tumor cells (Fig. 1E, Supplementary Figs. 1E, and 2E). Given this evidence, it was clear that the TME strongly modulated status of CRC patients.

**Figure 2:**
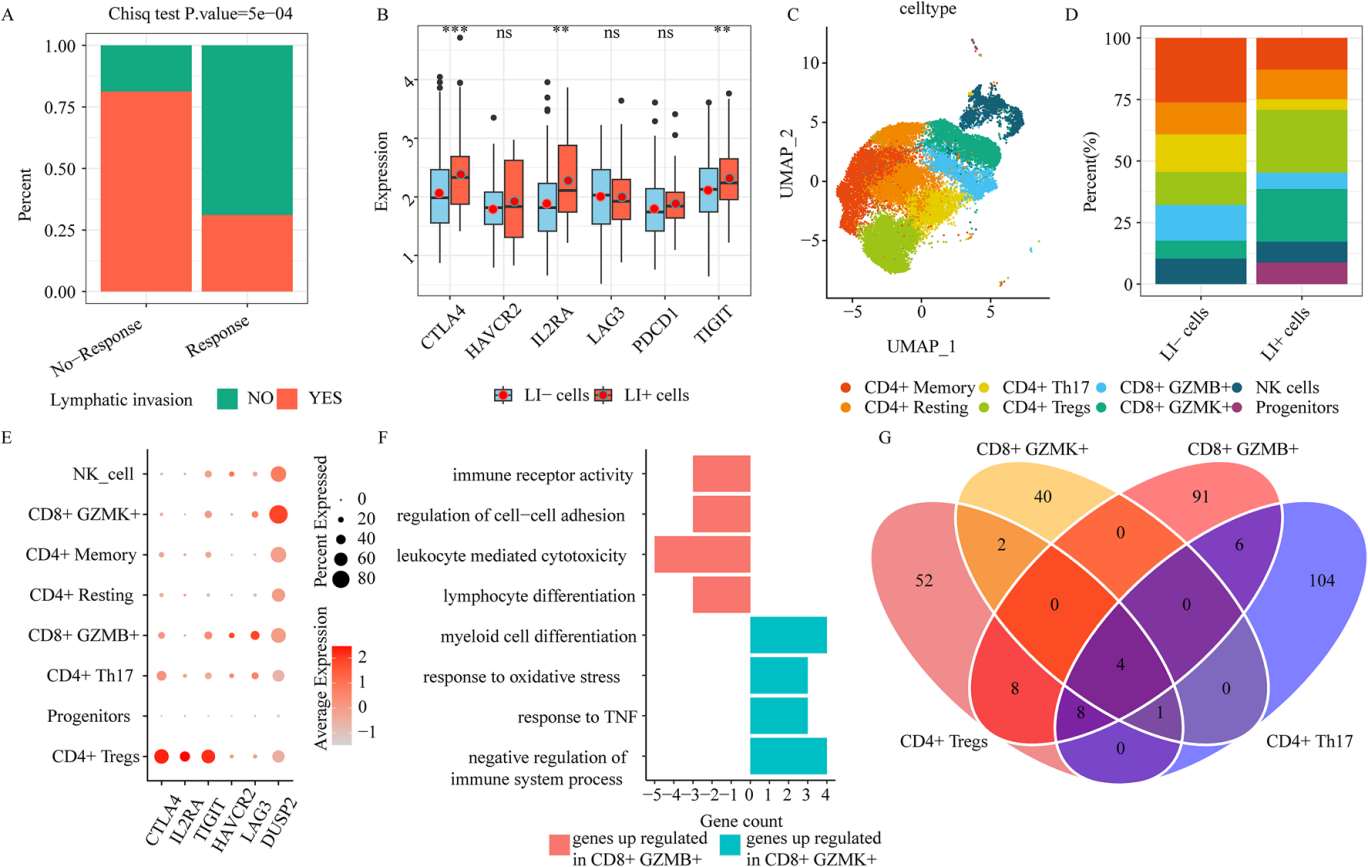
Immune response is diminished in lymphatic invasion (LI) patients. (**A**) Bar graph depicting the primary therapeutic response (complete/partial response (response), stable/progressive disease (no-response)) for LI and no-LI TCGA patients. (**B**) The immune checkpoint gene expression profiles between LI- and LI+ cells (ns ≥ 0.05, * < 0.05, ** < 0.01, *** < 0.001 and **** < 0.0001). (**C**) UMAP plots depicting all NK and T cells, colored according to cell sub-clusters. (**D**) Stacked bar chart depicting detailed components of individual NK/T cell clusters in LI- or LI+ cells. (**E**) The immune checkpoint gene expressions in 8 NK/T cell subclusters. (**F**) Enrichment analysis of upregulated genes in CD8+ GZMK+ or CD8+ GZMB+ cells. (**G**) The venn plot depicting DEG contents in CD4+ Tregs, CD8+ GZMK+, CD4+ Th, and CD8+ GZMB+, by comparing LI+ or LI-cells to the remaining cells.

**Figure 3:**
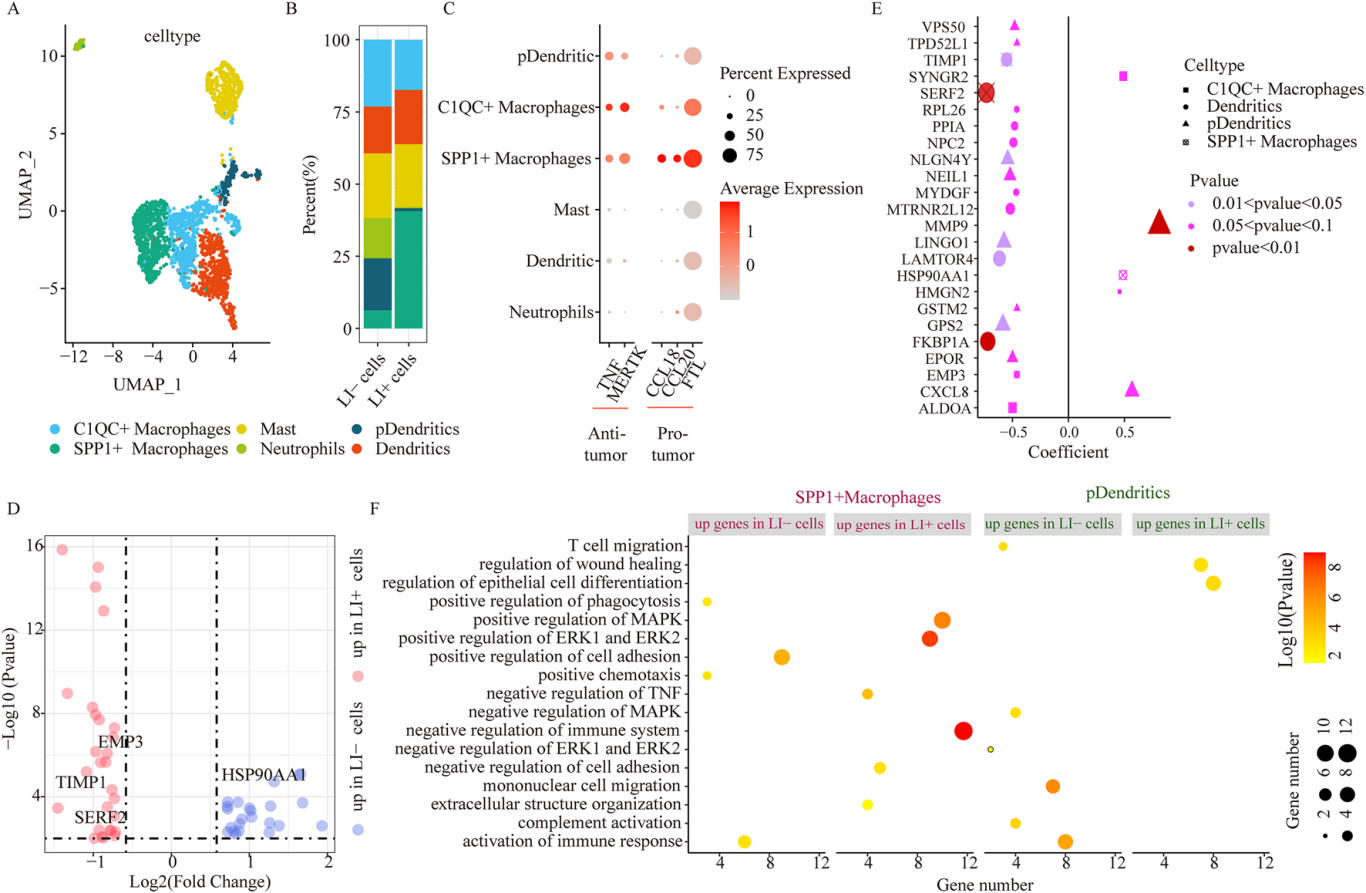
Myeloid cellular cluster comparison between LI+ and LI- cells. (**A**) UMAP plots depicting all myeloid cells, colored according to cellular sub-clusters. (**B**) Stacked bar chart illustrating the detailed compositions of individual myeloid cell clusters in LI- and LI+ cells. (**C**) The tumor suppressor and tumor promoting gene expressions in individual myeloid sub-clusters. (**D**) DEG evaluation via comparison of LI+ or LI-cells to the remaining cells in SPP1+ macrophages. (**E**) Association between gene expression and OS for DEGs via comparison among LI+ or LI-cells and the remaining cells in individual myeloid sub-clusters. Dot size represents the absolute correlation coefficient value, and shape indicates the cell subsets. (**F) **Enrichment analysis for DEGs via comparison of LI+ or LI-cells to the remaining cells in SPP1+ macrophages and pDCs.

**Figure 4:**
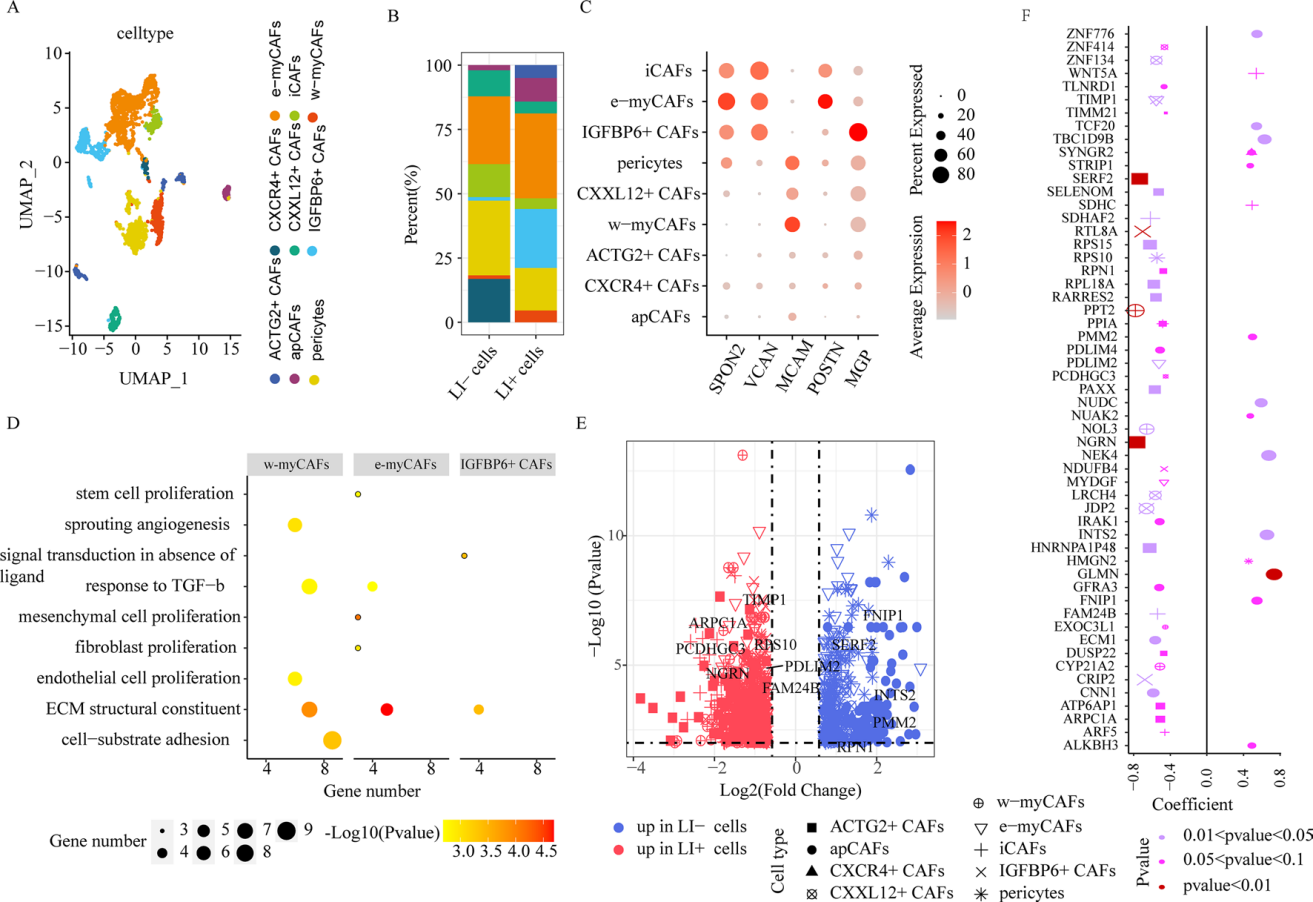
CAF cell cluster comparisons between LI+ and LI- cells. (**A**) UMAP plots depicting all CAF cells, colored according to the cell sub-clusters. (**B**) Stacked bar chart illustrating the detailed compositions of individual CAF cell clusters in LI- and LI+ cells. (**C**) The expression profiles of genes (SPON2, VCAN, MCAM, MGP, and POSTN) whose enhanced expression is correlated with disease progression and metastasis, in individual CAF sub-clusters. (**D**) Enrichment analyses of significantly upregulated genes in e-myCAFs, w-myCAFs, and IGFBP6+ CAFs. (**E**) DEG evaluation via comparison of the LI+ or LI-cells to the remaining cells in individual CAF cell clusters. (**F**) Association between gene expression and patient OS for DEGs via comparison of LI+ or LI-cells to the remaining cells in individual CAF sub-clusters. Dot size represents the absolute correlation coefficient value, and shape indicates the cellular subsets.

From the 3 aforementioned datasets, we respectively acquired 25,667, 38,233, and 17,713 cells from tumor tissues. Previous studies have revealed T^13^, myeloid^7^ and fibroblast^15^ cells played an important role in the progression of CRC. Thereafter, to better elucidate the T, myeloid and fibroblast profiles in various TME of CRC LI patients in greater detail, we combined these cells for tumor tissues from all 3 datasets, namely, NK/T, myeloid, and fibroblasts cell populations. Following batch effect correction, we conducted second stage clustering, and we obtained diversity of cell populations (Figs. 2C, 3A, and 4A). This showed that there were complex TMEs in CRC patients. Lastly, to explore the TMEs-mediated regulation of LI in CRC patients, we examined the link between cells and LI-specific phenotypes. Our findings from the aforementioned analyses are described in detail below (Figs. 2D, 3B, and 4B).

### Distinct CD8+ and CD4+ T cell states regulate the pro-invasive immune response

We retrieved the TCGA-COAD clinicopathological data (such as, lymphatic_invasion and primary therapy outcome success) from UCSC xena, which examined 164 LI patients and 250 no-LI patients, along with 195 complete response patients, 12 partial response patients, 25 progressive disease patients, and 4 stable disease patients. Our extensive analysis of LI and primary therapeutic success revealed that LI patients exhibited substantially reduced therapeutic response and experienced worse survival, relative to no-LI (Fig. 2A, Supplementary Fig. 3). To better elucidate the single-cell transcriptome data between LI and no-LI patients, we employed scissor to correspond single-cell data to LI-specific phenotypes via TCGA-COAD bulk RNA-seq sample. We revealed 513 LI-related (LI+ cells) and 641 no-LI-related (LI− cells) cells (Supplementary Fig. 4A). Notably, immune checkpoint genes, such as, CTLA4, IL2RA, and TIGIT were strongly differentially expressed (DE) between LI+ and LI− cells (Fig. 2B). To explore the role of T cells in promoting LI formation in CRC patients, we stratified NK/T cells into progenitors, NK cell, CD4+ T cell, and CD8+ T cell clusters (Fig. 2C, Supplementary Fig. 4B). Individual cell clusters exhibited contributions from distinct datasets (Supplementary Fig. 4C), which indicated no marked batch effects during clustering. Among the CD4+ T cell states, CD4+ Tregs (harboring FOXP3), CD4+ Memory (harboring CCR7, SELL, and TCF7), CD4+ Resting (harboring ANXA1), and CD4+ Th (harboring IL22 and IL17A) were recognized as 4 independent clusters (Supplementary Fig. 4B). Of note, we demonstrated that CD4+ Tregs were more enriched in LI+ than in LI− cells (Fig. 2D). We further validated this using CD4+ Tregs signature gene set scores via the TCGA-COAD bulk RNA-seq (Supplementary Fig. 4D). Additional assessment revealed that the immune checkpoint markers, CTLA4, IL2RA, and TIGIT, were relatively abundant in CD4+ Tregs (Fig. 2E). Based on these evidences, the tumor immune microenvironment (TIME) strongly contributes to shaping LI, which is, in turn, regulated by immune evasion during LI states in CRC patients.

Among the CD8+ T cell states, CD8+ GZMB+ and CD8+ GZMK+ were recognized as 2 independent CD8+ cytotoxic cell clusters (Fig. 2C, Supplementary Fig. 4B). These cellular states were previously reported in CRC patients^13^. CD8+ GZMK+, which possesses pro-inflammatory properties^16^, exhibited a higher population in LI+ cells, relative to the LI- cells (Fig. 2C), and this was further validated by CD8+ GZMK+ cell signature gene set scores using the TCGA-COAD bulk RNA-seq (Supplementary Fig. 4D). Alternately, CD8+ GZMB+ cells were enriched in LI− cells (Fig. 2C), which suggested a possible antagonistic function between CD8+ GZMK+ and CD8+ GZMB+ cells. Moreover, we observed no marked alterations in the CD8+ GZMB+ cell populations in the LI+ and LI− TCGA-COAD patients, which may be due to an underestimation of CD8+ GZMB+ cells infiltration abundance by bulk RNA-seq. To further elucidate the antagonistic property between CD8+ GZMK+ and CD8+ GZMB+ cells, we conducted gene expression and Gene Ontology (GO) term analysis for the 2 CD8+ subsets. We revealed that the DUSP2 (serving as a T cell suppressor to attenuate host antitumor immunity^17^) expression as well as the negative immune system regulatory pathways were substantially upregulated in CD8+ GZMK+ cells (Fig. 2E,F). In contrast, we revealed marked elevation in exhaustion marker expressions (LAG3, HAVCR2) as well as the leukocyte-driven cytotoxicity pathway in CD8+ GZMB+ cells (Fig. 2E,F). Collectively, these evidences confirmed that TIME strongly regulated LI, and CD8+ GZMK+ cells accelerated LI by weakening the immune response.

We next examined the transcriptome alterations in LI+ versus LI− states, and identified differentially expressed genes (DEGs) in CD4+ Tregs, CD8+ GZMK+, CD4+ Th, and CD8+ GZMB+ cells. Based on our observation, most DEGs were not common among T cell clusters (Fig. 2G, Supplementary Fig. 4E,F). This suggested that distinct molecular networks were activated among distinct T cell types to promote or inhibit LI.

### Myeloid subtyping and contribution to LI

Prior investigations also reported that myeloid cells critically regulated CRC progression^7^. Herein, we identified 2603 myeloid cells, which were sub-classified into 6 clusters, according to their canonical marker gene expression, and these included macrophages (harboring MARCO, CD68, TREM2 and MRC1; including 2 subsets), neutrophils (harboring CSF3R, S100A8 and S100A9), dendritic cells (harboring CD1C, CD1E, FCER1A and PKIB), pDCs (harboring DNASE1L3 and LAMP5) and mast cells (harboring MS4A2 and TPSAB1) (Fig. 3A,B and Supplementary Fig. 5A). We also identified two macrophage subcategories, namely, C1QC+ macrophages and SPP1+ macrophages using upregulated expressions of C1QC and SPP1 respectively. Additionally, chemokine genes CCL18^18^ and CCL20^19^ are reported to accelerate cancer progression via positive regulation of migration and angiogenesis, respectively, and these genes exhibited enhanced expression in SPP1+ macrophages (Fig. 3C). FTL^20^, which possesses both pro-proliferative and pro-angiogenic properties, was also upregulated in SPP1+ macrophages (Fig. 3C). In all our expression analysis supported that the SPP1+ macrophages controlled the pro-metastatic behavior of CRC, which corroborated with prior reports^7^. Nonetheless, the C1QC+ macrophages which have been reported to perform cytophagic and antigen-presenting function^7^, exhibited enhanced TNF and MERTK expressions, which may slow down tumor progression (Fig. 3C). Collectively, our evidences regarding the two macrophage subsets closely mirrored reports from earlier publication^7^ that existed the dichotomous functional phenotypes between SPP1+ macrophages and C1QC+ macrophages in the CRC TME^7^.

We next examined the association between relative abundance of various myeloid subtypes and LI-associated cells. Using scissor, we recognized 293 cells related to LI (LI+ cells), and 272 cells related to no-LI (LI− cells) (Supplementary Fig. 5B). More importantly, we discovered that the SPP1+ macrophage population was augmented in LI+ versus LI− cells. In contrast, we revealed that plasmacytoid dendritic cells (pDCs) were more prominent in LI− cells (Fig. 3B). Lastly, using bulk dataset integration, we also achieved the same conclusions (Supplementary Fig. 5C). We further explored the differing transcriptional distributions of SPP1+ macrophages and pDCs between LI+ cells and LI− cells.

In case of SPP1+ macrophages, 28 genes were highly expressed in LI+ cells, whereas, 23 were augmented in LI− cells (Fig. 3D). Of note, among the elevated genes in LI+ cells, we observed that the TIMP1 and SERF2 contents closely associated with poor survival of CRC patients (Fig. 3D,E). Additionally, we utilized the TCGA-COAD bulk samples to obtain the same conclusion that TIMP1 and SERF2 contents were elevated in LI patients (Supplementary Fig. 5D). Subsequently, we employed functional enrichment analysis to show that invasive networks, namely, negative immune modulation, negative TNF regulation, positive MAPK modulation, positive ERK1, ERK2, and extracellular matrix (ECM) regulation, were upregulated in the LI+ cells of SPP1+ macrophages (Fig. 3F). Alternately, among the elevated genes in LI− cells, we observed marked activation of immune response. In pDCs, 305 genes were augmented in LI+ cells, and 46 genes were augmented in LI− cells (Supplementary Fig. 5E). Among the elevated genes in LI+ cells, we found a strong inverse relationship between EPOR, GPS2, GSTM2, LINGO1, NEIL1, NLGN4Y, TPD52L1, and VPS50 gene expressions and patient survival (Fig. 3E). Among the elevated genes in LI-cells, the CXCL8 and MMP9 expressions were positively associated with patient survival (Fig. 3E). Using functional enrichment analysis, we further validated that the immune response-associated networks, namely, IL-17, TLRs, and TNF axes were strongly diminished in LI+ cells, in relation to LI− cells (Fig. 3F, Supplementary Fig. 5F). Together, these evidences indicated that the SPP1+ macrophage infiltration in LI+ cells accelerated LI, in turn pDCS infiltration in LI- cells, modulated anti-tumor immunity. Thus, targeting these signatures may diminish invasion and enhance patient clinical outcome.

### CAFs exert diverse functions in LI

Cancer-associated fibroblasts (CAFs) are critical contributors to the TME. Following re-clustering, we identified 9 fibroblast subsets with distinct properties, all of which were defined as CAFs owing to the expressions of angiogenesis- and immunomodulation-related genes (PDGFRA, PDGFRB, FAP, NOTCH3, HES4, THY1, CXCL12, CXCL14, CCL2, CXCR4, and ACTG2) (Supplementary Fig. 6). Subcluster cells that expressed antigen-presenting molecules, namely, HLA-DRA and HLA-DRB1 were designated as apCAFs (Fig. 4A, Supplementary Fig. 7A); cells that expressed collagen-associated genes, namely, COL1A1, COL5A1, and ATCA2 were referred to as myCAFs (Fig. 4A, Supplementary Fig. 7A); cells that expressed pericyte markers, namely, RGS5, PDGFRB, and CD248, were noted as pericytes (Fig. 4A, Supplementary Fig. 7A); and lastly, cells that expressed chemokines, namely, CXCL12, CXCL14, and CCL2, were recognized as iCAFs (Fig. 4A, Supplementary Fig. 7A). Among the myCAF cell states, e-myCAFs (harboring ECM molecules like MMP14 and LOXL2) and w-myCAFs (harboring contractile myofibroblast factors, namely, MYL9 and TAGLN) were designated as two independent clusters (Fig. 4A, Supplementary Fig. 7A). The remaining 4 subclusters were defined as CXCL12+ CAFs, CXCR4+ CAFs, ACTG2+ CAFs, and IGFBP6+ CAFs, respectively, based on their unique gene expressions. This reflected the CAF heterogeneity among CRC patients (Fig. 4A, Supplementary Fig. 7A).

Till date, there are no single-cell level studies on CAF heterogeneity and association with LI in CRC patients. Using scissor, we identified 218 cells as LI+ cells, due to their marked association with LI, and 148 cells as LI− cells owing to their marked association with no-LI (Supplementary Fig. 7B). Importantly, we revealed that both e-myCAFs and w-myCAFs were more abundant in LI+ versus LI− cells (Fig. 4B). Following a combined analysis of bulk datasets, we also obtained the same conclusion that the e-myCAF and w-myCAF cell abundance was substantially upregulated in LI versus no-LI patients (Supplementary Fig. 7C). Moreover, using enrichment analysis of significantly upregulated e-myCAF and w-myCAF genes, we revealed that both cells expressed elevated levels of proliferation- and ECM remodeling network-related genes, whereas, w-myCAFs also showed enrichment for angiogenesis-associated genes (Fig. 4D). Based on these evidences, the upregulated e-myCAFs and w-myCAFs in CRC patients likely enhance invasion and metastasis. Furthermore, we revealed that invasion-related gene expression SPON2^21^, VCAN^22^, and POSTN^23^ was heavily upregulated in e-myCAFs, whereas, MCAM was augmented in w-myCAFs (Fig. 4C). Collectively, these results suggested that both e-myCAFs and w-myCAFs enhance invasion and metastasis^24^. Moreover, we revealed that a previously unreported CAF subpopulation (harboring IGFBP6, a gene strongly associated with VSMC physiological function^25^) (Fig. 4B), was more enriched in LI+ cells than in LI− cells. Lastly, following a combined analysis of bulk datasets, we obtained the comparable conclusions (Supplementary Fig. 7C). Additionally, we revealed that the MGP expression, a gene that is inversely related to patient prognosis, was substantially elevated in IGFBP6+ CAFs (Fig. 4C). To assess possible function of augmented IGFBP6+ CAF abundance in CRC patients, we conducted pathway enrichment analysis, and revealed that the ECM and signal transduction networks in absence of ligand were remarkably enriched (Fig. 4D). Collectively, these evidences indicated that IGFBP6+ CAFs could accelerate metastasis and modulate prognosis of CRC patients.

Furthermore, the apCAF population, which are known to regulate immune evasion in pancreatic cancer^26^, was also remarkably enhanced in LI+ versus LI− cells, and this was further validated using apCAF signature gene set scores via TCGA-COAD-based bulk RNA-seq (Supplementary Fig. 7C). The apCAFs are also considered to be antigen presenting cells. Thus, we analyzed its association with T cells. We revealed that, in the SPP1 axis-based interactions, apCAFs with enhanced SSP1 expressions exhibited more cellular crosstalk with T cells with enhanced CD44 expression (Supplementary Fig. 7D). Since it has been early reported that the SPP1-CD44 ligand-receptor pair causes immunosuppression in intrahepatic cholangiocarcinoma progression^27^, we speculated that the augmented apCAF abundance in CRC patients may potentially induce immune escape. Collectively, these evidences suggested that the heterogeneous CAFs exerted multiple functions to form LI, including accelerating invasion and enhancing immune escape.

### Establishment of 3 subcategories in CRC patients using invasive genes

**Figure 5:**
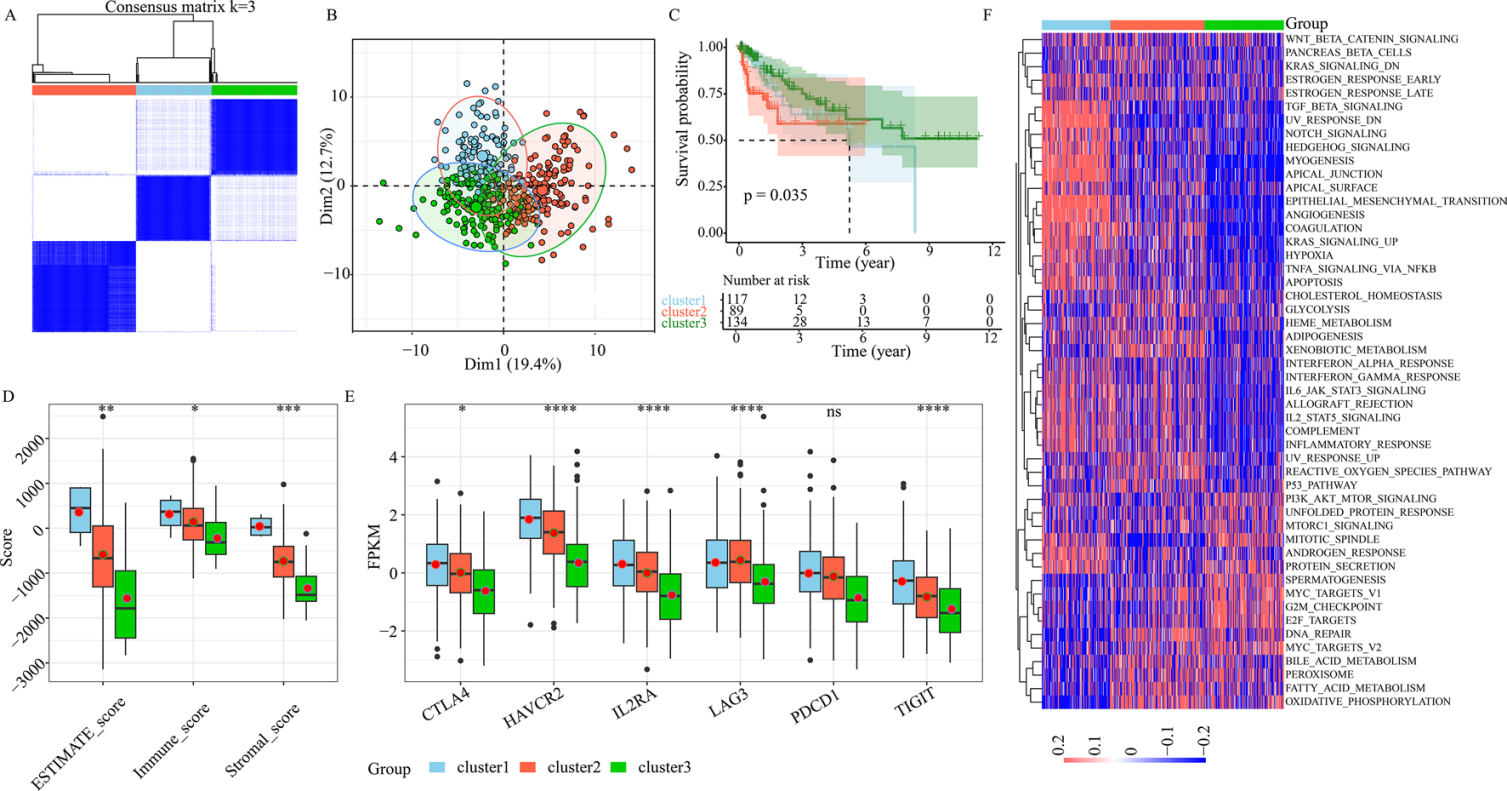
Consensus clustering of lymphatic invasion (LI)-associated genes in TCGA-COAD. (**A**) Consensus matrices of TCGA patients. (**B**) PCA analysis of the 3 subgroups in TCGA cohort. (**C**) KM curves depicting prognosis of the 3 TCGA subgroups. (**D**) Stromal, immune, and estimate scores among the 3 subgroups (ns ≥ 0.05, * < 0.05, ** < 0.01, *** < 0.001 and **** < 0.0001). (**E**) The immune checkpoint gene expressions among 3 subgroups (ns ≥ 0.05, * < 0.05, ** < 0.01, *** < 0.001 and **** < 0.0001). (**F**) Heatmap depicting gene set variation analysis scores of the 50 hallmark gene sets in the 3 subgroups of colorectal cancer (CRC). Color intensity represents scores.

Herein, we employed single-cell and TCGA analysis to evaluate the TME infiltration status of CRC patients, and confirmed our identification of the LI-related cells from single-cell data with phenotypic guidance from bulk data. This information can potentially enhance cell-targeted therapies and identification of robust prognostic markers. Through our comparison of LI+ or LI− cells with all other cells from individual cellular subset, we conducted extensive DE (Fig. 4E) and overall survival (OS) analysis. Our conclusions were as follows: 60 genes were substantially elevated in LI+ cells, and had negative correlation with patient OS. Moreover, 19 genes were enhanced in LI− cells, showed positive correlation with patient OS (Figs. 3E and 4F, and Supplementary Fig. 4F). Additionally, our unsupervised clustering analysis of 79 gene expressions from the TCGA-COAD dataset revealed 3 distinct regulatory patterns. These included 129 cases in cluster 1, 178 cases in cluster 2, and 148 cases in cluster 3 (Fig. 5A). We next separated all participants into 3 cohorts, based on the PCA results, and confirmed the presence of 3 distinct subtypes (Fig. 5B). Based on our survival analysis, the 3 newly segregated subtypes exhibited markedly different prognosis (Fig. 5C, *P* < 0.05), with the cluster 3 patients experiencing the best prognosis.

In addition, we performed an extensive assessment of clinicopathological profiles among the 3 distinct subtypes. We observed no discernible differences in the tumor stage, gender, age and stage among the 3 subtypes (Supplementary Fig. 8). However, the cluster 3 patients exhibited the highest proportion of no-LI (Supplementary Fig. 8). To better elucidate differences in immune response, we compared the immune scores of various subtypes. We revealed that cluster 3 had the lowest immune cell and stromal cell scores, whereas, cluster 1 produced the largest immune cell and stromal cell scores (Fig. 5D). We next compared the profiles of immune checkpoint molecules, namely, PD-1, CTLA4, LAG3, TIGIT, IL2RA, and HAVCR2. We revealed that the cluster 3 patients produced the lowest expressions of immune checkpoint molecules (Fig. 5E). Lastly, we demonstrated significant differences in the carcinogenic profiles of the 3 clusters using GSVA analysis with the 50 Hallmark gene sets. Cluster 1 showed enrichment in the EMT networks, namely, TGF-B, NOTCH, and epithelial mesenchymal transition axes (Fig. 5F). Cluster 2 showed enrichment in metabolism networks, namely, GLYCOLYSI and XENOBIOTIC (Fig. 5F). Cluster 3 showed enrichment in proliferation networks, namely, E2F TARGETS, G2M CHECKPOINT, and MYC TARGETS (Fig. 5F). Based on these evidences, we identified certain LI targets, which modulate tumor development, metastasis and immune response, and have great potential in enhancing prognosis of CRC patients.

### Development and validation of the LI-related prognostic model

**Figure 6:**
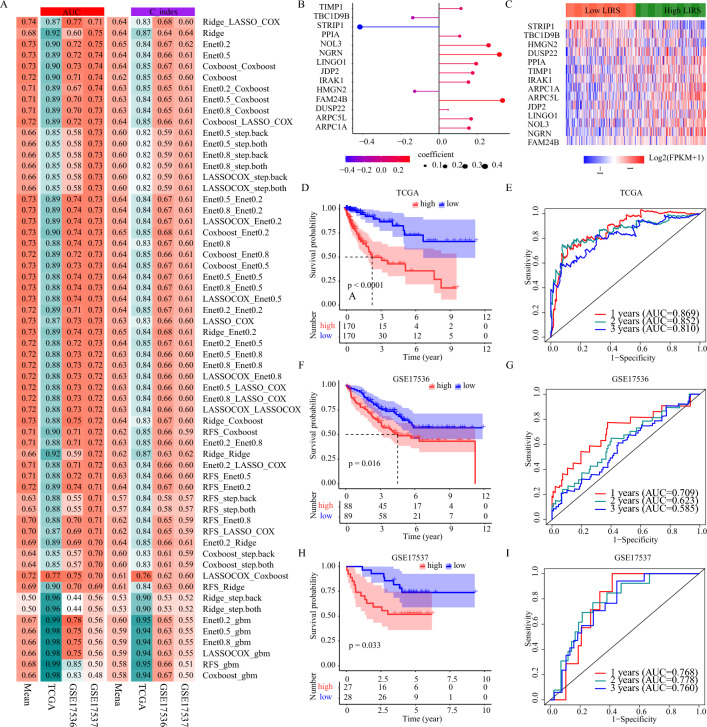
Development and validation of the LI-related prognostic model. (**A**) The AUC and C-indexes of 60 machine-learning algorithm combinations in the TCGA-COAD training cohort and the two testing cohorts. (**B**) Coefficients of the 14 LI-related signatures in the cox regression model. (**C**) The differential expression of 14 LI-related signatures between high- and low-LIRS subgroups based on median level of LIRS in TCGA-COAD. (**D**–**I**) Kaplan–Meier survival curve of OS between high- and low-LIRS, and ROC curves at 1, 2 and 3 years in the TCGA-COAD training cohort and the two testing cohorts.

To develop an ideal biomarker for exactly stratifying the prognosis, based on these above-mentioned LI targets, we applied 60 machine-learning algorithm combinations to construct prediction models in the TCGA-COAD training cohort, and calculated the mean AUC and C-index of each algorithm in the two testing cohorts (GSE17536, GSE17537). As shown in Fig. 6A, the combination of Ridge (genes, with the coefficient > 0.01, were selected) and LASSO Cox with the highest average AUC (0.74) was selected as the final model. As illustrated in Fig. 6B, the LI-related risk score (LIRS) was developed according to the expression of 14 LI-related signatures with following equation: LIRS = (0.168 ∗ ARPC5L) + (0.260 ∗ NOL3) + (0.115 ∗ TIMP1) + (0.334 ∗ FAM24B) + (0.108 ∗ PPIA) + (0.157 ∗ ARPC1A) + (0.317 ∗ NGRN) + (0.047 ∗ DUSP22) + (0.155 ∗ IRAK1) + (− 0.416 ∗ STRIP1) + ( − 0.138 ∗ TBC1D9B) + (0.177 ∗ JDP2) + ( − 0.128 ∗ HMGN2) + (0.195 ∗ LINGO1). The expression differences of the 14 LI-related signatures between high and low LIRS groups according to the median value in TCGA-COAD patients was shown in Fig. 6C. To evaluate the prognostic performance of LIRS, the Kaplan–Meier curve of OS demonstrated the high LIRS group possessed significantly shorter survival time in the TCGA-COAD training cohort (*p* < 0.0001 Fig. 6D). The time-dependent ROC curves at 1, 2 and 3 years of OS with the AUC values of 0.869, 0.852 and 0.810 confirmed the prognostic value of the LIRS in the TCGA-COAD training cohort (Fig. 6E). The LIRS was further independently validated in the two testing cohorts. The patients with high LIRS group possessed significantly shorter survival time both in the GSE17536 cohort and in the GSE17537 cohort (*p* < 0.05 Fig. 6F,H), and the AUC values of 1, 2 and 3 years were 0.709, 0.623, and 0.585 in the GSE17536 cohort, 0.768, 0.778, and 0.760 in the GSE17537 cohort (Fig. 6G,I). The above results further clarified the LIRS could produce an accurate prognostic prediction, and we provided valuable mechanistic insights into LI in clinical process.

## Discussion

LI is more aggressive and produces worse prognosis in CRC patients^2^. Thus, it is crucial to characterize the cellular and molecular pathways of LI, and recognize new robust targets for improvement of CRC patient prognosis. Herein, we provided a high-resolution view for dissecting the TME in CRC by integrating 230,302 single cells from 35 patients across 3 datasets. Then we integrated scRNA-seq and TCGA LI-specific phenotypic data for CRC patients to investigate the TME of LI versus no-LI patients, and provided a comprehensive perspective for dissecting the cellular and molecular mechanisms in the LI -related TME, further our results were confirmed via TCGA bulk RNA-seq data. Our single-cell analysis revealed that the LI+ cells exhibited enhanced expression of immune checkpoint genes, as well as an augmented abundance of CD4+ Tregs (an immunosuppressive subset) and CD8+ GZMK+ cells. CD8+ GZMK+ cells in CRC patients expressed large quantities of DUSP2, and genes of negatively regulate immune networks. The CD8+ GZMK+ cells in CRC patients have been reported to be correlated with worse outcome^16^. These suggested that the quantity of infiltrating CD4 Tregs and CD8+ GZMK+ cells could form the suppressive TME and affected therapeutic response and prognosis of LI patients in CRC. Prior investigations also reported that myeloid cells critically regulated CRC progression. Zhang et al. identified the dichotomy of C1QC+ macrophages and SPP1+ macrophages in CRC, namely C1QC+ macrophages was reported to perform cytophagic and antigen-presenting function, whereas SPP1+ macrophages was reported to perform pro-angiogenic and tumor-promoting functions, and the patients with high SPP1+ and low C1QC+ macrophages signatures had the poor prognosis^7^. Lee et al.^28^ also showed that expansion of SPP1+ macrophages may perform immune suppression and tumor promotion in CRC patients, and patients with high SPP1 expression had a poorer prognosis. In our paper, we also identified the 2 independent macrophage populations in CRC, namely, C1QC+ and SPP1+ macrophages, and interestingly, we observed the abundance of SPP1+ macrophages was largely enhanced in LI+ cells. The SPP1 has been reported to be a key gene of lymph node metastasis both in lung adenocarcinoma^29^ and head and neck carcinoma^30^. Dong et al. identified a higher abundance of macrophages in the lymph node metastasis tumor of lung adenocarcinoma^29^ using the immunohistochemistry. Based on these previous researches, we preliminarily speculated that the SPP1+ macrophages promoted LI and induced poor prognosis in CRC. Meanwhile we also observed the most reduced abundance of pDCs in LI+ cells. The elevated LI+ or LI− cellular genes in SPP1+ macrophages and pDCs respectively, revealed that the TNF and immune response were severely diminished in LI+ cells. Together, these evidences about the T and myeloid cells suggested that the immune response was strongly inhibited in LI TME, which corroborated with prior reports of immunosuppression contributing to distal metastasis^31,32^. These findings may have important implications for improving cancer immunotherapy in CRC LI patients, such as the combination therapies that target both T and myeloid cells.

CAFs strongly promote tumor metastasis in numerous cancer types^33^. Prior investigations identified multiple CAF subpopulations among CRC patients^33^, however, the molecular mechanisms underlying CAF function in the CRC LI TME remain poorly determined. Herein, we revealed that LI+ cells exhibited enhanced e-myCAF, w-myCAF, and apCAF infiltration. Both e-myCAF and w-myCAF subclusters revealed enhanced expressions of invasion-related genes. Meanwhile, both ECM- and angiogenesis-related networks were also substantially upregulated in the two subclusters. In case of the apCAF subcluster, we identified strong immunosuppressive interactions between apCAFs and T cells in CRC patients. Interestingly, we recognized a previously unreported CAF subpopulation (IGFBP6+ CAFs), which showed augmented abundance in LI+ cells, and MGP, which was inversely associated with patient OS, was specifically upregulated in this CAF subcluster. In all, in this report, we demonstrated that the heterogeneous CAFs served diverse roles that developed LI.

In conclusion, herein, we assessed TME differences between LI+ and LI- at a single-cell level. We also demonstrated that LI+ cells exhibited augmented abundance of immunosuppression and invasive subset, relative to LI- cells. The TME encompasses a complicated ecosystem that enhances LI. Thus, a combinational therapy targeting ≥ 2 TME subclusters may be a promising strategy to combat invasion and enhance patient OS. We observed marked ECM network activation in LI+ cells within SPP1+ macrophages, as well as in augmented abundance CAFs subclusters for LI+ cells. This suggested that specific targeting of the ECM networks may effectively suppress LI in CRC patients. In addition, we found some genes were associated with both LI and survival, and we employed immune scores, immune checkpoint molecule expression, and GSVA analysis to identify these genes which could impact tumor development, metastasis, and immune response in CRC patients. Finally, a novel LIRS model was established based on the expression of 14 LI-related signatures, and in the two testing cohorts LIRS was also proved to have accurate prognostic predictive ability, which further demonstrated the clinical importance of these targets. The conclusions from our study can be a valuable resource for an enhanced comprehension of the underlying pathways that contribute to LI. This information can potentially aid in the development of highly efficacious therapeutic targets and biomarkers for CRC patient OS enhancement.

## Materials and methods

### Data source and processing

RNA-seq from COAD patient samples were acquired from TCGA using the TCGA biolinks R package. To analyze the gene expression data, we collected FPKM data, a normalized estimation of gene expression according to the RNA‐seq data. In all, we acquired 3 scRNA-seq transcriptome datasets of CRC patients from the GEO database, namely, GSE200997, GSE166555, and GSE201348.

### scRNA-seq data preprocessing

We utilized the R package Seurat (v4.4.0)^34^ to convert that way matrix count for individual sample. Subsequently, we removed genes expressed in < 3 cells. Low-quality cells were eliminated using parameters as follows: Cells containing < 500 UMIs, or < 200 expressed genes, or > 20% mitochondrial content. Using LogNormalize, we confirmed equal quantities of total gene expression profile of individual cells, and the scale factor was adjusted to 10,000. Then, we employed the FindVariableFeatures function to identify the leading 2000 DEGs. We next used principal component analysis (PCA) to minimize dataset dimensionality, and the leading 30 PCs were chosen for UMAP. Finally, we utilized FindClusters function to recognize cell clusters. Batch effect correction utilized Seurat cca during 3 dataset integration. Cell identity annotations on individual clusters were designated according to the expressions of established canonical marker genes. Cell subclusters with comparable gene expression profiles were then designated as the same cell type. All aforemetioned analyses were conducted with the R package Seurat (v4.4.0).

### DEGs identification and signaling network analysis

Highly expressed genes of each cell subcluster, as well as DEGs between LI+ or LI- cells and other cell types in each cell subset, were recognized via the “FindAllMarker” function in Seurat using parameters: |logFC|> 0.7 and only.pos = TRUE. DEGs or biomarker genes were selected using Wilcoxon Rank Sum test, based on a threshold value (*p*-value < 0.05). The clusterProfiler (4.6.2) R package was employed for GO and KEGG^35–37^ network enrichment analysis. Lastly, we verified TME components in CRC patients, using the R package GSVA (1.46.0), in combination with the ssGSEA algorithm, for quantification of the relative abundance of individual cellular invasion in the TCGA-COAD bulk sample.

### OS analysis

RNA-seq and COAD patient clinical information were acquired from TCGA for evaluation of prognostic influence of genes. Individual gene expressions were determined via the log2 (FPKM+ 1) scale. Single factor Cox regression analysis was employed for DEGs analysis. Lastly, OS curves were fitted via the Kaplan–Meier formula in R survival package (v3.5-7).

### CRGs consensus clustering analysis

Our comparison of LI+ or LI− cells to all other cells in each cell subset yielded DEGs. We next conducted univariate Cox analysis on the newly identified DEGs to screen for prognostic-associated genes with *p*-value < 0.05. We discovered 73 prognosis-related genes. Using the ConsensusClusterPlus (1.62.0) R package, we clustered patients according to the profiles of 73 aforementioned genes in tumor tissues from the TCGA dataset. Next, we utilized the FactoMineR (2.9) package to conduct PCA analysis. To next assess the clinical performance of the invasive subtypes, we examined the associations between invasive subtypes and patient prognosis as well as other clinicopathological characteristics, namely, patient age, gender, stage, grade, and treatment response.

### Cell–cell crosstalk

We assessed cell-to-cell crosstalk using CellChat (1.6.1). We utilized single cell gene expression matrix and corresponding cellular type to elucidate potential association strength between individual cell type. The cell-to-cell crosstalk was further evaluated using communication probability and *p*-value from the output file (*p* ≤ 0.01).

### Gene set variation analysis (GSVA)

To estimate the distinct signaling network enrichment scores among the 3 distinct molecular subgroups, we conducted GSVA via GSVA (1.46.0) R package^38^. Gene sets were obtained from the MsigDB database.

### Development the LI-related prognostic model

To construct a consensus prognosis model, we integrated 8 classical algorithms, random forest (RFS), LASSO COX, GBM, ridge regression, CoxBoost, Stepback Cox, Stepboth Cox, and elastic network (Enet), into 60 machine-learning algorithm combinations. We utilized the TCGA-COAD as the training cohort, and the GSE17536 and GSE17537 as the testing cohort. Finally, we calculated the mean AUC and C-index of each algorithm in the two testing cohorts, and picked the best consensus LIRS model based on the average.

### Statistical analysis

All data analyses were performed on the R software. Categorical data were assessed using the chi-square test and continuous data were analyzed using the Wilcoxon rank sum test. *P* < 0.05 was adjusted as the significance threshold.

### Supplementary Information

Supplementary Figure 1.

Supplementary Figure 2.

Supplementary Figure 3.

Supplementary Figure 4.

Supplementary Figure 5.

Supplementary Figure 6.

Supplementary Figure 7.

Supplementary Figure 8.

Supplementary Legends.

Supplementary Legends.

Supplementary Tables.

## Data Availability

The TCGA-COAD cohort can be downloaded from TCGA (https://portal.gdc.cancer.gov/) and datasets GSE200997, GSE166555, and GSE201348 are available in the NCBI GEO (https://www.ncbi.nlm.nih.gov/geo/) public repositoriy. In addition, we provided all the relevant source data in the Supplementary Tables.
