# Supplementary figures and images for: Single-cell transcriptome analysis profiling lymphatic invasion-related TME in colorectal cancer
Source: Sci Rep. 2024 Apr 17;14:8911. doi: 10.1038/s41598-024-59656-6 (PMC11024122; doi:10.1038/s41598-024-59656-6)

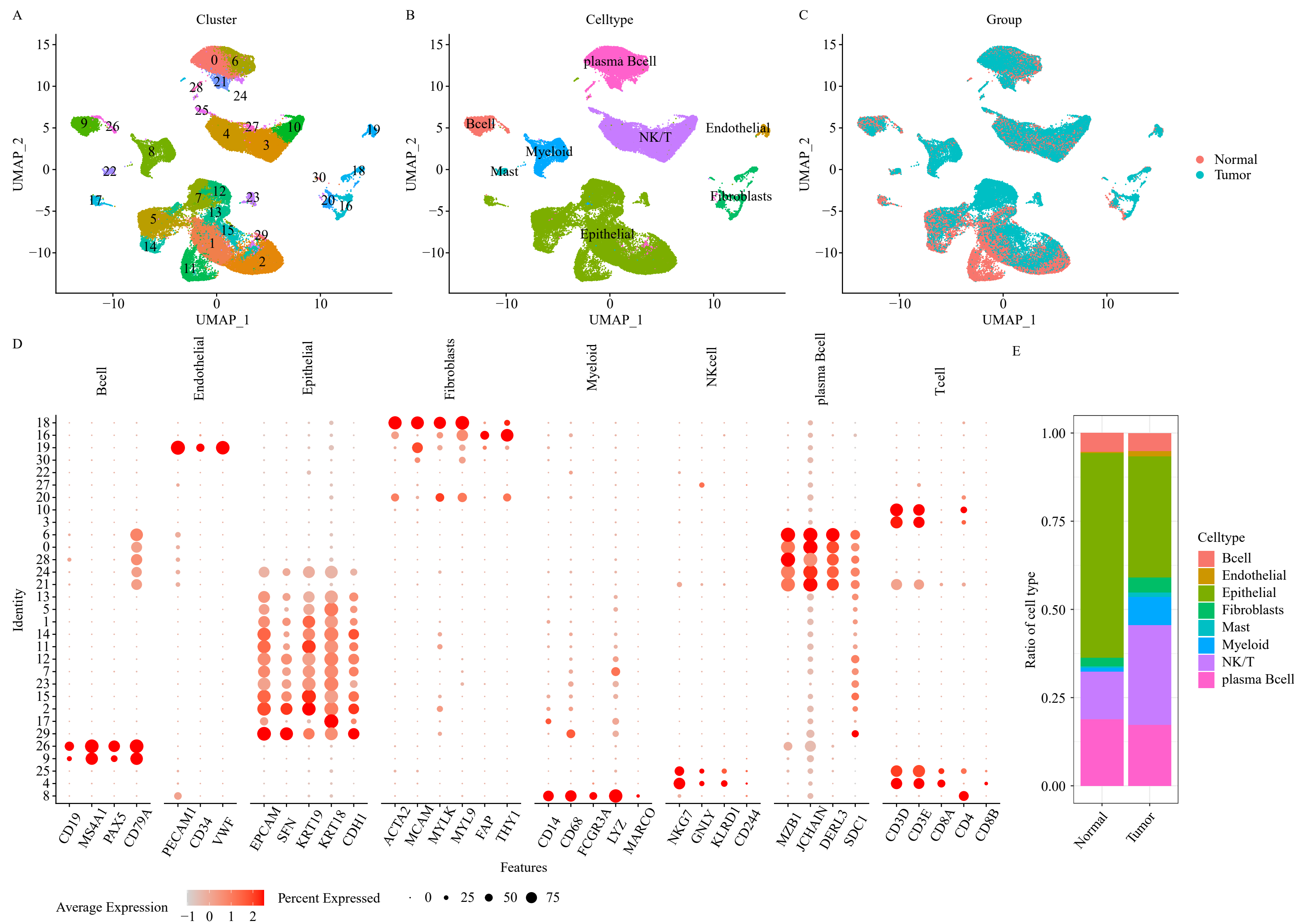

Supplement: Supplementary file 1 — Supplementary Figure 1. [file 41598_2024_59656_MOESM1_ESM.pdf]

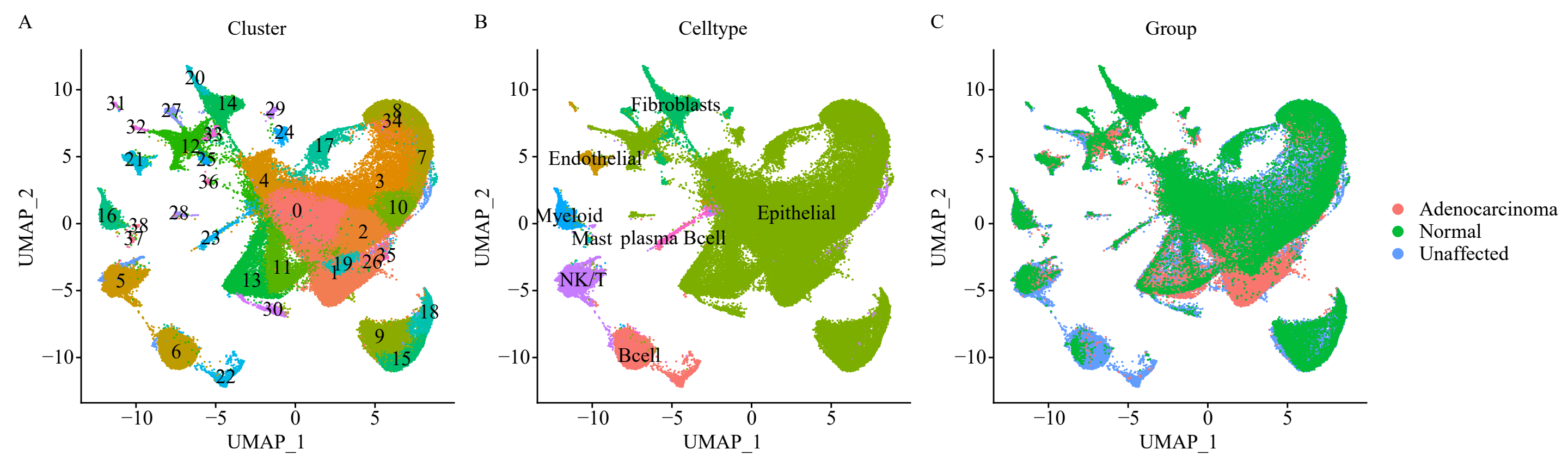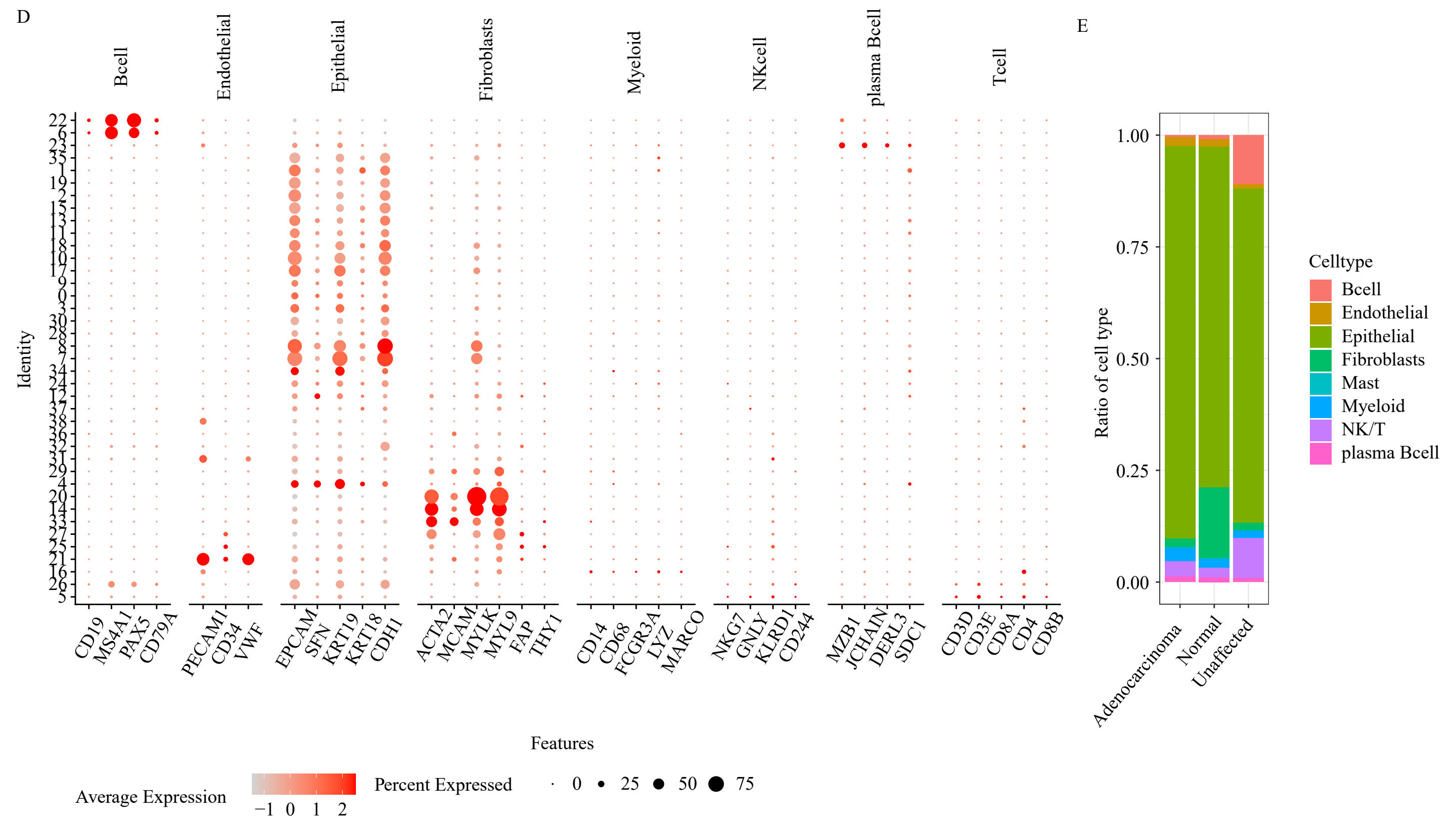

Supplement: Supplementary file 2 — Supplementary Figure 2. [file 41598_2024_59656_MOESM2_ESM.pdf]

**Supplementary Figure 3.** KM curves of TCGA cohorts.

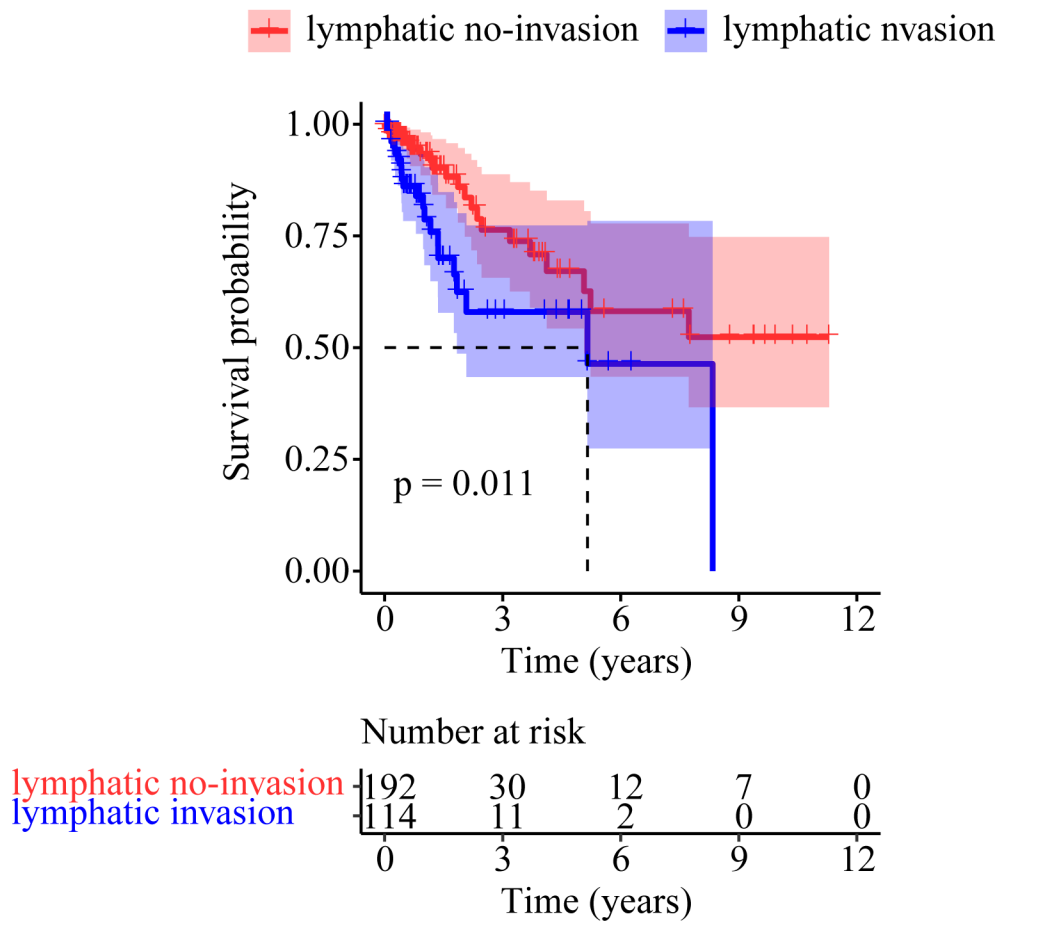

Supplement: Supplementary file 3 — Supplementary Figure 3. [file 41598_2024_59656_MOESM3_ESM.pdf]

Supplementary Figure 4. Characteristics of NK/T cell sub-clusters.

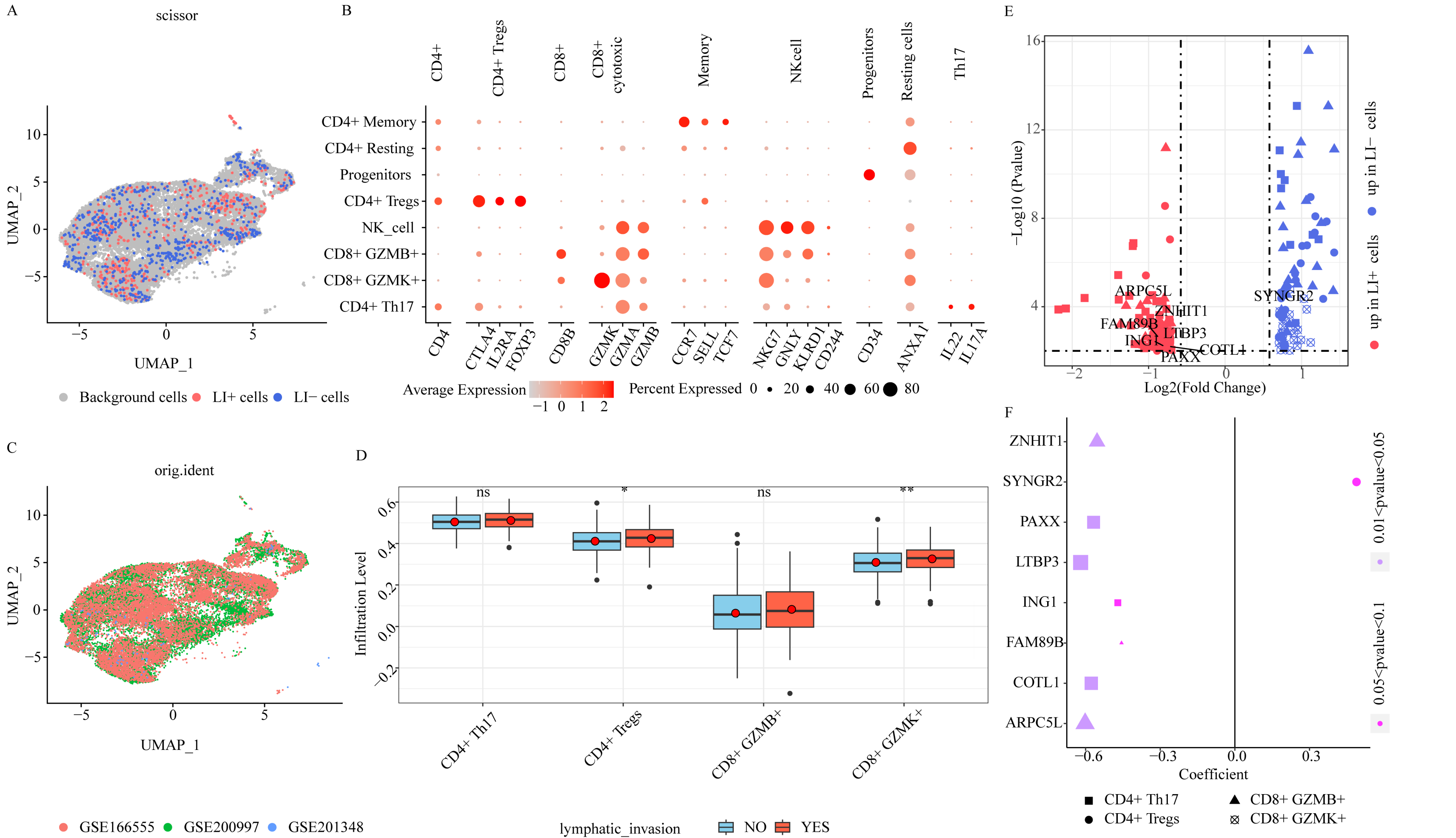

Supplement: Supplementary file 4 — Supplementary Figure 4. [file 41598_2024_59656_MOESM4_ESM.pdf]

Supplementary Figure 5. Characteristics of Myeloid cell sub-clusters.

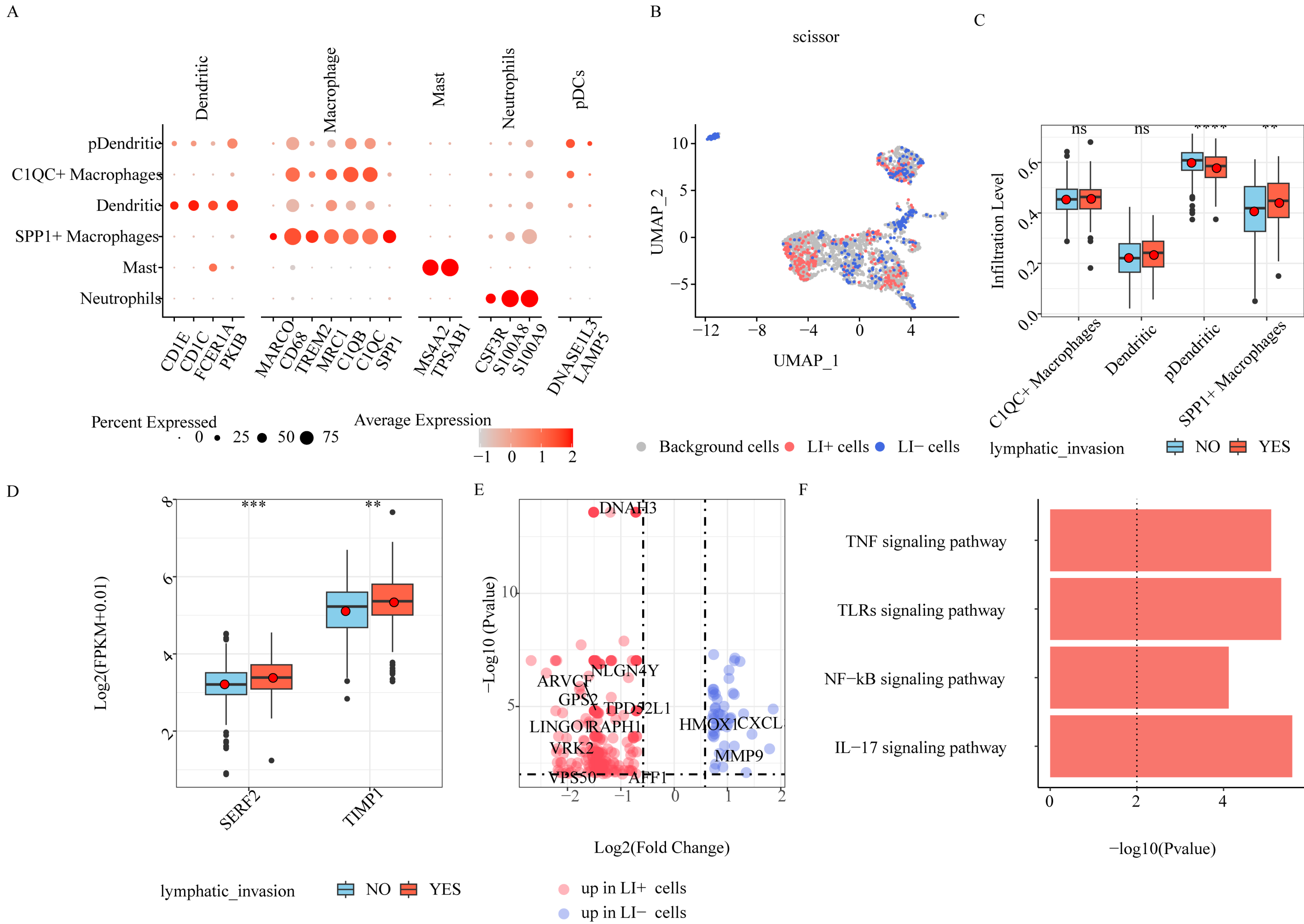

Supplement: Supplementary file 5 — Supplementary Figure 5. [file 41598_2024_59656_MOESM5_ESM.pdf]

Supplementary Figure 7. Characteristics of CAF sub-clusters.

A

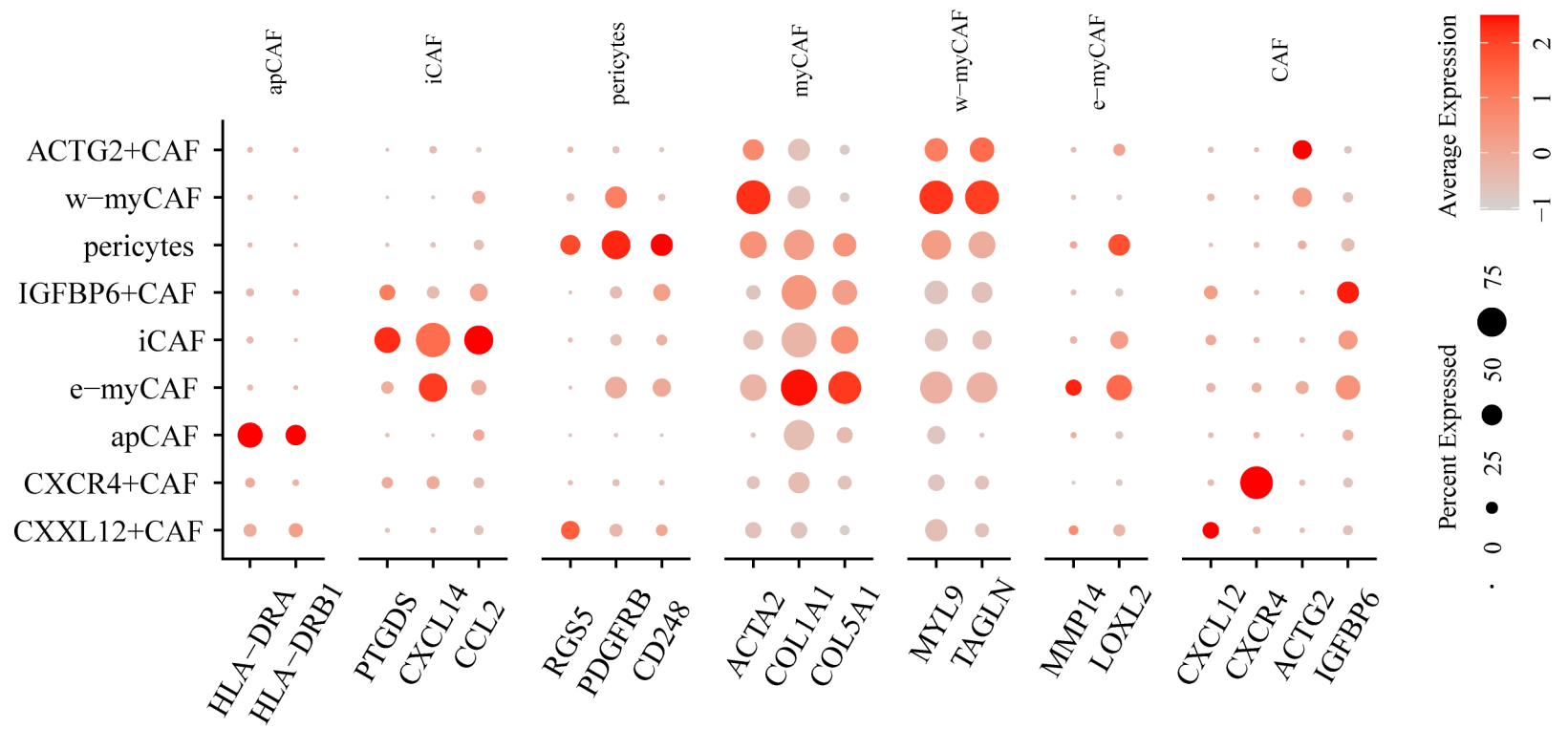

B

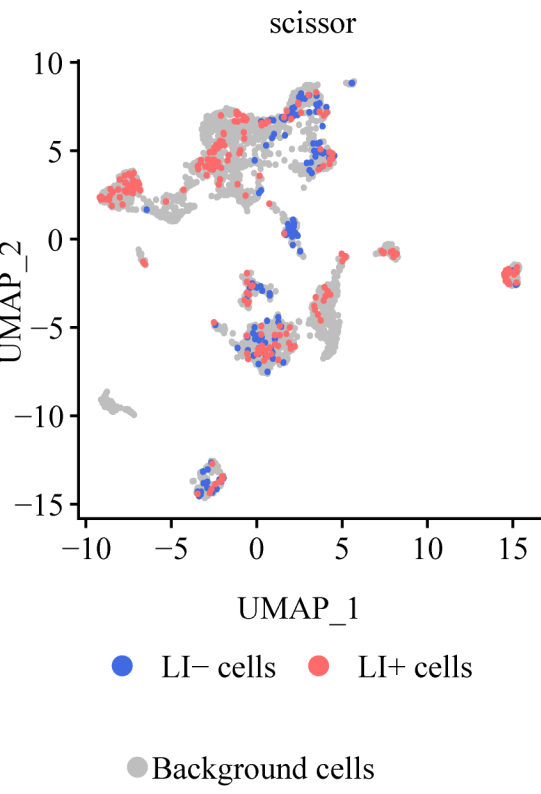

C

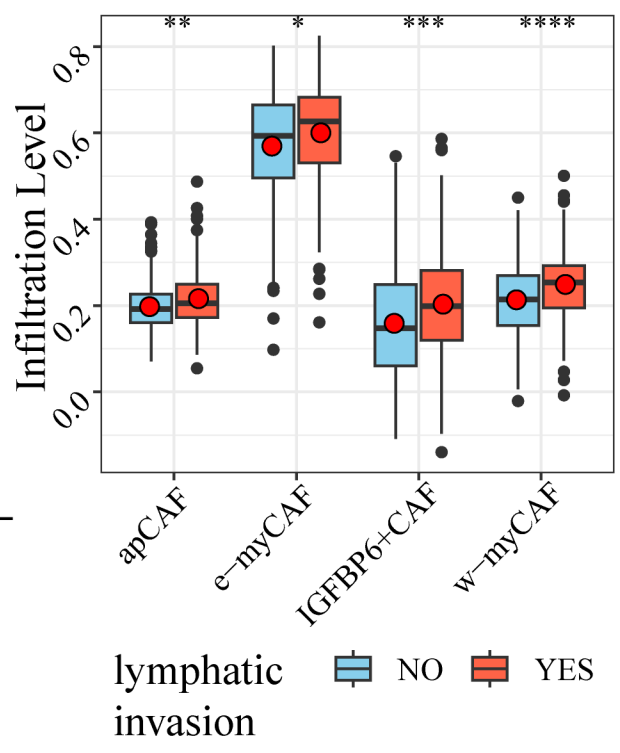

D

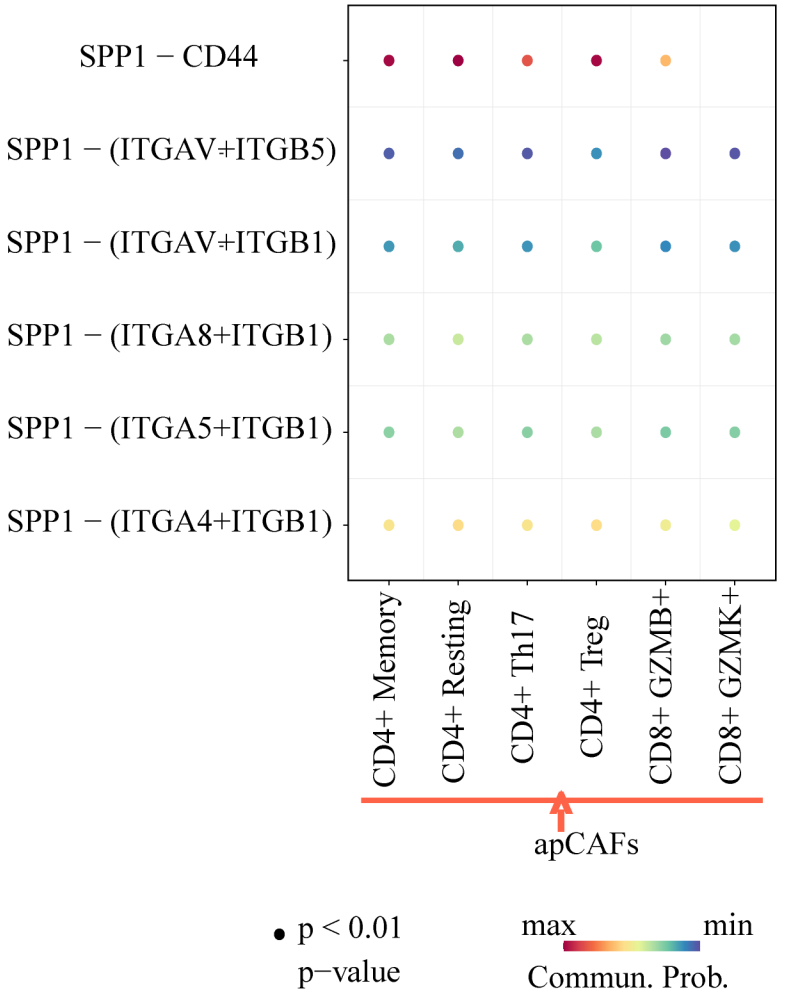

Supplement: Supplementary file 7 — Supplementary Figure 7. [file 41598_2024_59656_MOESM7_ESM.pdf]

**Supplementary Figure 8.** The difference of clinical features in the three subgroups.

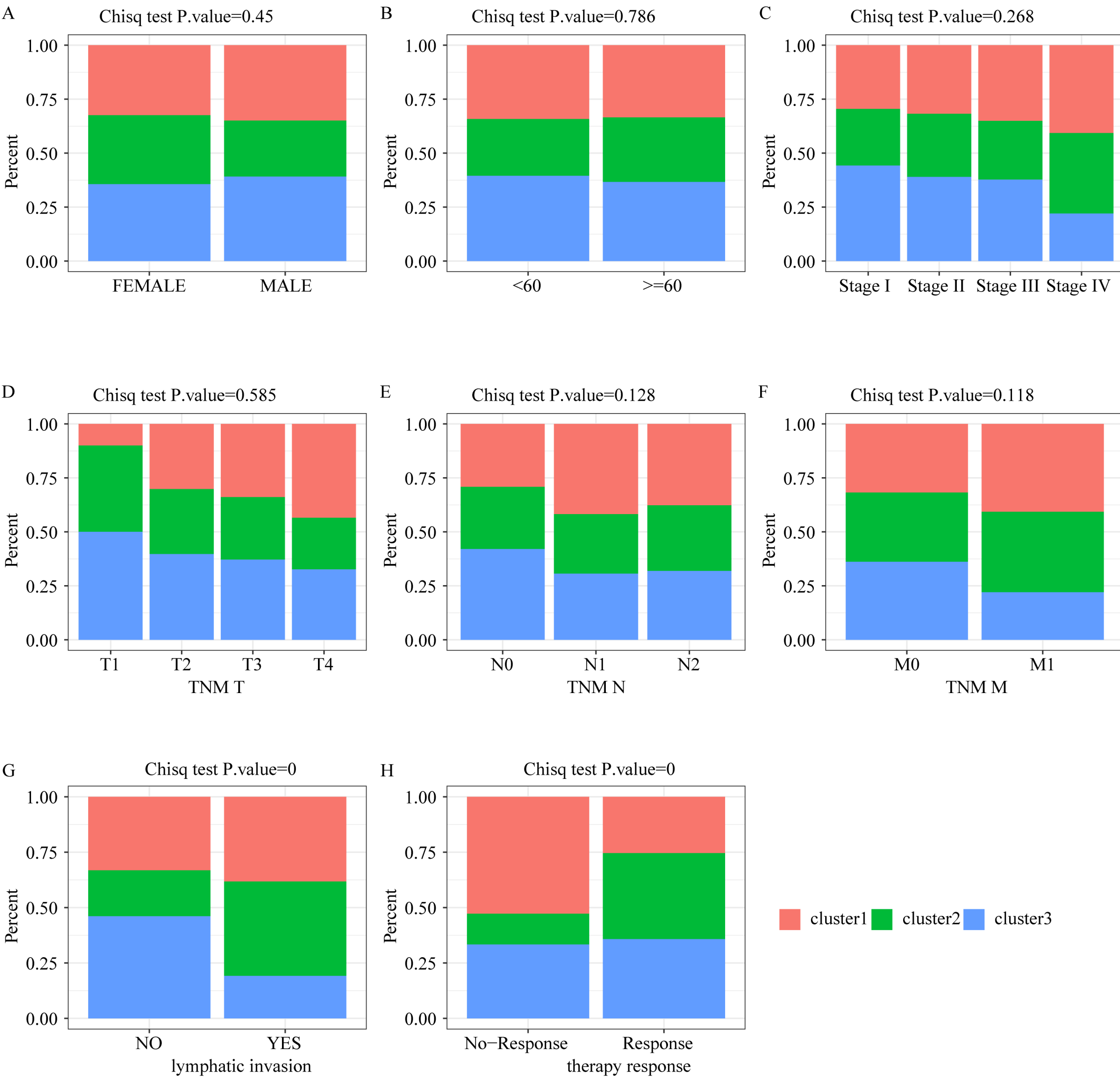

Supplement: Supplementary file 8 — Supplementary Figure 8. [file 41598_2024_59656_MOESM8_ESM.pdf]
