# Supplementary material for: Single-cell transcriptome analysis profiling lymphatic invasion-related TME in colorectal cancer
Source: Sci Rep. 2024 Apr 17;14:8911. doi: 10.1038/s41598-024-59656-6 (PMC11024122; doi:10.1038/s41598-024-59656-6)

Supplementary Figure 6. The expression of angiogenesis- and immunomodulation-related genes in 9 fibroblasts subsets.

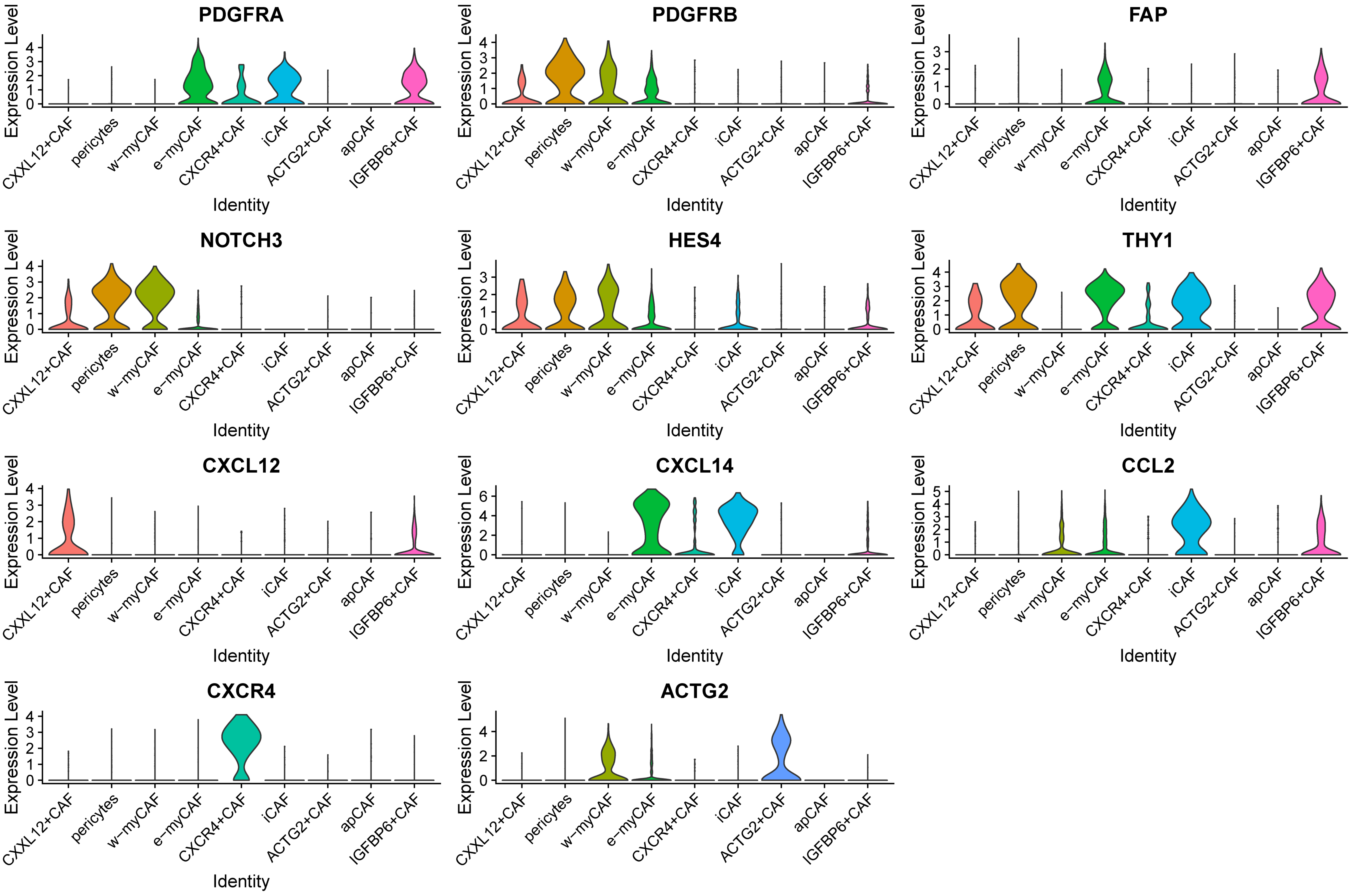

Supplement: Supplementary file 6 — Supplementary Figure 6. [file 41598_2024_59656_MOESM6_ESM.pdf]
