# Supplementary material for: Single-cell transcriptome analysis profiling lymphatic invasion-related TME in colorectal cancer
Source: Sci Rep. 2024 Apr 17;14:8911. doi: 10.1038/s41598-024-59656-6 (PMC11024122; doi:10.1038/s41598-024-59656-6)
Supplement: Supplementary file 9 — Supplementary Legends. [file 41598_2024_59656_MOESM9_ESM.docx]

***Supplementary Figure Legends***

**Supplementary Figure 1.** A summary of the single cells in CRC patients, and recognition of primary cell types in the GSE166555 dataset. (A) UMAP plot depicting single cells (colored according to cell cluster). (B) UMAP plot depicting single cells (colored according to cellular type). (C) UMAP plot depicting single cells (colored according to sample origins, either tumor versus normal samples). (D) Dot plot illustrating representative marker genes across all cellular clusters. Dot size indicates fraction of specific gene-expressing cells. Color intensity indicates relative specific gene expressions. (E) Stacked bar chart depicting 7 major cellular type contents in individual tumor or normal samples.

**Supplementary Figure 2**. A summary of the single cells in CRC patients, and recognition of primary cell types in the GSE201348 dataset. **(A)** UMAP plot depicting single cells (colored according to cell cluster). **(B)** UMAP plot depicting single cells (colored according to cellular type). **(C)** UMAP plot depicting single cells (colored according to sample origins, either tumor versus normal samples). **(D)** Dot plot illustrating representative marker genes across all cellular clusters. Dot size indicates fraction of specific gene-expressing cells. Color intensity indicates relative specific gene expressions. **(E)** Stacked bar chart depicting 7 major cellular type contents in individual tumor or normal samples.

**Supplementary Figure 3**. KM curves demonstrating the different prognosis between the LI and no-LI patients in TCGA cohorts.

**Supplementary Figure 4**. Characteristics of NK/T cell sub-clusters. **(A)** UMAP plot of the scissor-selected cells (the red and blue dots are cells associated with the LI and no-LI phenotypes, respectively). **(B)** Dot plot of mean expression of canonical marker genes for the eight cell types. Dot size is proportional to the fraction of cells expressing specific genes. Color intensity corresponds to the relative expression of specific genes. **(C)** UMAP plot of the analyzed single cells (colored by from the three datasets). (**D)**The infiltration of CD4+ Tregs, CD4+ Th17, CD8+ GZMB+ and CD8+ GZMK+ between the LI and no-LI patients in TCGA cohorts. The circle dot represents mean values. *P < 0.05, **P < 0.01, ***P < 0.001, ****P < 0.0001, ns, not significant (two-sided unpaired Wilcoxon test). **(E)** The DEGs by comparing the LI+ cells, LI-cells to the other cells respectively in CD4+ Tregs, CD8+ GZMK+, CD4+ Th and CD8+ GZMB+ cells. **(F)** The correlation between gene expression and survival for DEGs by comparing the LI+ cells, LI-cells to the other cells respectively in CD4+ Tregs, CD8+ GZMK+, CD4+ Th and CD8+ GZMB+ cells, dot size is the absolute value of correlation coefficient, shape corresponds to the cell subsets.

**Supplementary Figure 5**. Characteristics of Myeloid cell sub-clusters. **(A)** Dot plot of mean expression of canonical marker genes for the six cell types. Dot size is proportional to the fraction of cells expressing specific genes. Color intensity corresponds to the relative expression of specific genes. **(B)** UMAP plot of the Scissor-selected cells (The red and blue dots are cells associated with the LI and no-LI phenotypes, respectively). **(C)** The infiltration of Dendritic, pDendritic, C1QC+ Macrophages and SPP1+ Macrophages between the LI and no-LI patients in TCGA cohorts. The circle dot represents mean values. *P < 0.05, **P < 0.01, ***P < 0.001, ****P < 0.0001, ns, not significant (two-sided unpaired Wilcoxon test). **(D)** The expression of SERF2 and TIMP1 between the LI and no-LI patients in TCGA cohorts. **(E)** The DEGs by comparing the LI+ cells, LI-cells to the other cells in pDCs. **(F)** The KEGG enrichment analysis for specifically overexpressing genes respectively by comparing the LI-cells to the other cells in pDCs.

**Supplementary Figure 6**. The expression of angiogenesis- and immunomodulation-related genes in 9 fibroblasts subsets.

**Supplementary Figure 7**. Characteristics of CAF sub-clusters. **(A)** Dot plot of mean expression of canonical marker genes for the nine cell types. Dot size is proportional to the fraction of cells expressing specific genes. Color intensity corresponds to the relative expression of specific genes. **(B)** UMAP plot of the scissor-selected cells (The red and blue dots are cells associated with the LI and no-LI phenotypes, respectively). **(C)** The infiltration of apCAFs, e-myCAFs, w-myCAFs and IGFBP6+CAFs between the LI and no-LI patients in TCGA cohorts. The circle dot represents mean values. *P < 0.05, **P < 0.01, ***P < 0.001, ****P < 0.0001, ns, not significant (two-sided unpaired Wilcoxon test). **(D)** The significant ligand-receptor pairs for the SPP1 signaling pathway network that contribute to the signaling sending from apCAFs to the Tcell subsets. The dot color and size represent the calculated communication probability and p-values. p-values are computed from one-sided permutation test.

**Supplementary Figure 8**. The difference of clinical features in TCGA cohort in the three subgroups by Chi-square test.
