# Supplementary material for: Single-cell transcriptome analysis profiling lymphatic invasion-related TME in colorectal cancer
Source: Sci Rep. 2024 Apr 17;14:8911. doi: 10.1038/s41598-024-59656-6 (PMC11024122; doi:10.1038/s41598-024-59656-6)
Supplement: Supplementary file 10 — Supplementary Legends. [file 41598_2024_59656_MOESM10_ESM.docx]

**Supplementary Tables** **Content**

Supplementary Table1. The identification of major cell types for datasets from GSE200997

Supplementary Table2. The identification of major cell types for datasets from GSE166555

Supplementary Table3. The identification of major cell types for datasets from GSE201348

Supplementary Table4. The constitution of seven main cell types in each tumor or normal samples from GSE200997

Supplementary Table5. The constitution of seven main cell types in each tumor or normal samples from GSE166555

Supplementary Table6. The constitution of seven main cell types in each tumor or normal samples from GSE201348

Supplementary Table7. The clinical data of TCGA patients

Supplementary Table8. The expression of immune checkpoint molecules between LI- cells and LI+ cells in NK and T cells

Supplementary Table9. The identification of cell subcluster for NK and T cells

Supplementary Table10. The detailed constitutions for each NK/T cell sub-cluster in LI- cells or LI+ cells

Supplementary Table11. The expression of immune checkpoint molecules in 8 NK/T cell sub-cluster

Supplementary Table12. Enrichment analysis of upregulated genes in CD8+ GZMK+ or CD8+ GZMB+ cells

Supplementary Table13. The DEGs in CD4+ Th17 between LI- and LI+ cells

Supplementary Table14. The DEGs in CD4+ Tregs between LI- and LI+ cells

Supplementary Table15. The DEGs in CD8+ GZMB+ between LI- and LI+ cells

Supplementary Table16. The DEGs in CD8+ GZMK+ between LI- and LI+ cells

Supplementary Table17. The expression of canonical marker genes for the eight NK/T cell types

Supplementary Table18. The infiltration of CD4+ Tregs, CD4+ Th17, CD8+ GZMB+ and CD8+ GZMK+ between the LI and no-LI patients in TCGA cohorts

Supplementary Table19. The genes significantly associated with survival for DEGs between LI+ cells and LI-cells in CD4+ Tregs, CD8+ GZMK+, CD4+ Th and CD8+ GZMB+ cells

Supplementary Table20. The identification of cell subcluster for Myeloid cells

Supplementary Table21. The tumor suppressor and tumor promoting gene expressions in individual myeloid sub-clusters

Supplementary Table22. The DEGs between LI+ and LI-cells in SPP1+ macrophages

Supplementary Table23. The genes significantly associated with survival for DEGs between LI+ cells and LI-cells in individual myeloid sub-clusters

Supplementary Table24. The enrichment analysis of DEGS between LI+ and LI-cells in SPP1+ macrophages and pDCs

Supplementary Table25. The expression of canonical marker genes for the six myeloid cell types

Supplementary Table26. The infiltration of Dendritic, pDendritic, C1QC+ Macrophages and SPP1+ Macrophages between the LI and no-LI patients in TCGA cohorts

Supplementary Table27. The expression of SERF2 and TIMP1 between the LI and no-LI patients in TCGA cohorts

Supplementary Table28. The DEGs between LI+ and LI-cells in pDendritics

Supplementary Table29. The KEGG enrichment analysis for specifically overexpressing genes respectively for LI-cells in pDendritics

Supplementary Table30. The identification of cell subcluster for Fibroblasts

Supplementary Table31. The detailed constitutions for each Fibroblasts sub-cluster in LI- cells or LI+ cells

Supplementary Table32. The expression profiles of genes (SPON2, VCAN, MCAM, MGP and POSTN) in individual Fibroblasts sub-clusters

Supplementary Table33. The enrichment analyses of significantly upregulated genes in e-myCAFs, w-myCAFs, and IGFBP6+CAFs

Supplementary Table34. The DEGs between LI+ and LI-cells in individual CAFs cell sub-cluster

Supplementary Table35. The genes significantly associated with survival for DEGs between LI+ cells and LI-cells in individual CAF sub-cluster

Supplementary Table36. The expression of angiogenesis- and immunomodulation-related genes in 9 fibroblasts subsets

Supplementary Table37. The expression of canonical marker genes for the nine CAF cell sub-clusters

Supplementary Table38. The infiltration of CAF sub-clusters between the LI and no-LI patients in TCGA cohorts

Supplementary Table39. The sub-group of TCGA patients by Consensus clustering of lymphatic invasion (LI)-associated genes

Supplementary Table40. Stromal, immune, and estimate scores among the 3 subgroups

Supplementary Table41. The immune checkpoint gene expressions among 3 subgroups

Supplementary Table42. The gene set variation analysis scores for the 50 hallmark gene sets in the TCGA patients

Supplementary Table43. The Survival information for 3 subgroups

Supplementary Table44. The download information for the raw data
